# Genetic and process engineering strategies for enhanced recombinant *N*-glycoprotein production in bacteria

**DOI:** 10.1186/s12934-021-01689-x

**Published:** 2021-10-14

**Authors:** Fenryco Pratama, Dennis Linton, Neil Dixon

**Affiliations:** 1 Manchester Institute of Biotechnology (MIB), The University of Manchester, Manchester, M1 7DN UK; 2 Department of Chemistry, The University of Manchester, Manchester, M1 7DN UK; 3 Faculty of Biology, Medicine and Health, The University of Manchester, Manchester, M1 7DN UK; 4 Microbial Biotechnology Research Group, School of Life Sciences and Technology, Institut Teknologi Bandung, Bandung, 40132 Indonesia

**Keywords:** Glycoengineering, *N*-glycosylation, Bacterial host engineering, Protein folding, Process optimisation

## Abstract

**Background:**

The production of N-linked glycoproteins in genetically amenable bacterial hosts offers great potential for reduced cost, faster/simpler bioprocesses, greater customisation, and utility for distributed manufacturing of glycoconjugate vaccines and glycoprotein therapeutics. Efforts to optimize production hosts have included heterologous expression of glycosylation enzymes, metabolic engineering, use of alternative secretion pathways, and attenuation of gene expression. However, a major bottleneck to enhance glycosylation efficiency, which limits the utility of the other improvements, is the impact of target protein sequon accessibility during glycosylation.

**Results:**

Here, we explore a series of genetic and process engineering strategies to increase recombinant N-linked glycosylation, mediated by the Campylobacter-derived PglB oligosaccharyltransferase in *Escherichia coli*. Strategies include increasing membrane residency time of the target protein by modifying the cleavage site of its secretion signal, and modulating protein folding in the periplasm by use of oxygen limitation or strains with compromised oxidoreductase or disulphide-bond isomerase activity. These approaches achieve up to twofold improvement in glycosylation efficiency. Furthermore, we also demonstrate that supplementation with the chemical oxidant cystine enhances the titre of glycoprotein in an oxidoreductase knockout strain by improving total protein production and cell fitness, while at the same time maintaining higher levels of glycosylation efficiency.

**Conclusions:**

In this study, we demonstrate that improved protein glycosylation in the heterologous host could be achieved by mimicking the coordination between protein translocation, folding and glycosylation observed in native host such as *Campylobacter jejuni* and mammalian cells. Furthermore, it provides insight into strain engineering and bioprocess strategies, to improve glycoprotein yield and titre, and to avoid physiological burden of unfolded protein stress upon cell growth. The process and genetic strategies identified herein will inform further optimisation and scale-up of heterologous recombinant *N*-glycoprotein production.

**Supplementary Information:**

The online version contains supplementary material available at 10.1186/s12934-021-01689-x.

## Background

Attachment of carbohydrates to proteins is the most abundant post-translational modification [1–3]. Protein glycosylation occurs in all Domains of life, and in eukaryotes more than half of all proteins are predicted to be glycosylated [4, 5]. It generally involves the transfer of glycans onto the amide side chain of asparagine (N-linked) or the hydroxyl group of serine or threonine (O-linked) amino acid residues. This extra layer of molecular information affects a variety of protein features, from structural dynamics such as folding and stability, to involvement in complex cellular physiology such as cell interactions and pathogenicity [6–9].

**Fig. 1:**
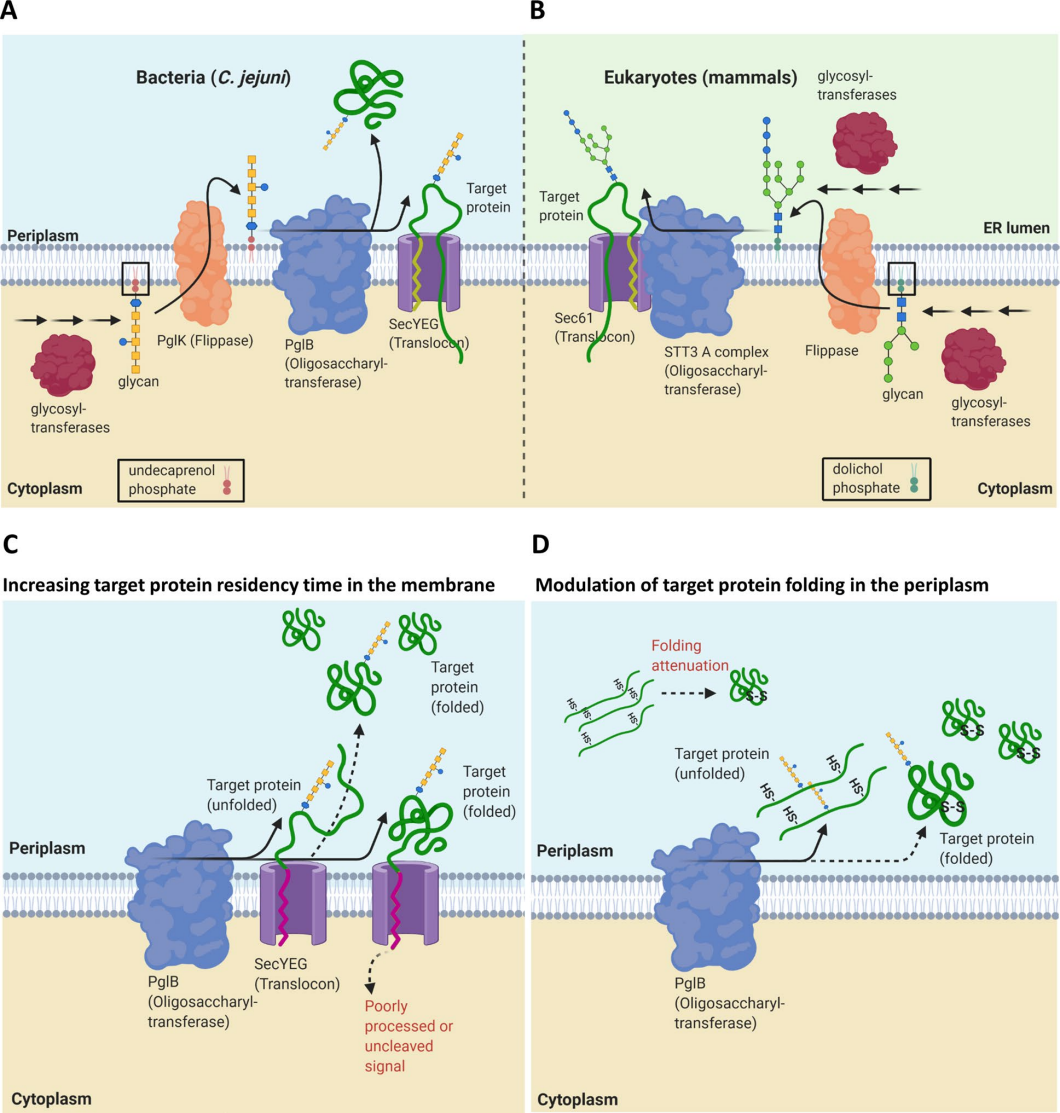
Schematic overview of native N-linked protein glycosylation pathway and proposed strategies in this study to improve sequon accessibility of recombinant target protein to PglB during glycosylation in glycoengineered *E. coli*. N-linked glycosylation in bacteria/*C. jejuni* (**A**) compared to early-stage of eukaryotic/mammalian N-linked glycosylation pathway (**B**). **A** In *C. jejuni*, undecaprenol-linked glycan is synthesised by glycosyltransferases in the cytoplasm, flipped to the periplasm by flippase (PglK), and then transferred co or post-translocationally to a target protein by PglB. **B** In mammalian N-linked glycosylation, dolichol-linked glycan is synthesised both in the cytosol and endoplasmic reticulum (ER) lumen, and glycan is transferred co- or post-translocationally (later not shown) by different STT3 isoforms. **C**, **D** Two different strategies are proposed to enhance heterologous protein glycosylation in *E. coli*. **C** Approach based on increasing PglB interaction with target protein in the membrane. Increased membrane residence of target protein was achieved by introducing signal peptide mutations with poor processivity or uncleaved signal. **D** Approach based on increasing sequon accessibility of target proteins to PglB via modulation of target protein folding state during or after translocation. Using a disulphide-bonded model protein, protein folding and maturation in the periplasm are delayed by producing the protein under suboptimal conditions for disulphide formation such as under oxygen-depleted conditions, in the absence of oxidoreductases, or under chemical redox treatment. Solid arrow = increased reaction, dashed arrow = reduced reaction

Eukaryotes and a small number of bacterial species from the epsilon subdivision of the Proteobacteria share a homologous process by which they perform N-linked protein glycosylation [10]. Both require assembly of a glycan on a lipid anchor, known as a lipid-linked oligosaccharide (LLO), followed by transfer of the synthesised glycan *en bloc* by an oligosaccharyltransferase (OTase) onto the asparagine residue of the acceptor protein. However, there are some key variations in reaction components and pathway locations (Fig. 1A, B) [11–13]. In eukaryotes, N-linked glycosylation is initiated on the cytosolic side of the rough endoplasmic reticulum (ER) membrane (Fig. 1B). There, glycosyltransferases (GTs) assemble a conserved eukaryotic heptasaccharide structure on a polyprenol diphosphate moiety known as dolichol. This LLO is then flipped to the luminal face of the ER and a further seven sugars are added by different GTs in the ER lumen before the glycan is transferred to the target protein by the OTase complex. In prokaryotes, the best characterised N-linked glycosylation pathway is *pgl* (protein glycosylation) in the bacterium *Campylobacter jejuni* (Fig. 1A) [11, 14, 15]. In this pathway, a *Cj* heptasaccharide structure is sequentially assembled by specific GTs onto an undecaprenol diphosphate lipid carrier on the cytosolic face of the inner membrane (IM), flipped onto the periplasmic face of the IM, and transferred to the target protein. A conserved enzyme in eukaryotic and prokaryotic N-linked glycosylation is the OTase, which catalyses covalent attachment of the glycan to the acceptor sequon of the target protein [16, 17]. The consensus acceptor sequon in Archaea and Eukaryota is N-X-S/T (X ≠ Proline), while in bacteria, acidic amino acids at the -2 position are required (D/E-X_1_-N-X_2_-S/T, X_1, 2_ ≠ Proline) [18, 19]. Except for some single-celled protists such as *Leishmania major* and *Trypanosoma brucei*, eukaryotic OTases are hetero-oligomeric, comprised of seven to nine proteins [10, 20]. The catalytic sub-unit of eukaryotic OTase is STT3 and in mammals two STT3 isoforms (STT3A and B) have distinct roles during N-linked glycosylation. The STT3A-dependent complex associates with the translocation machinery to optimise co-translational glycan transfer to protein, whilst STT3B-dependent glycosylation occurs following translocation [21, 22]. Bacterial N-linked OTases, such as PglB from *C. jejuni*, consist of a single transmembrane protein homologue of eukaryotic STT3, that can carry out both co- and post-translocational glycosylation [23–26].

Benefiting from improved pharmacological quality, about 70% of marketed recombinant human therapeutic proteins contain (mostly N-linked) glycans [27–29]. Production in mammalian cells, predominantly in the Chinese Hamster Ovary (CHO) cell, enables the coupling of complex mammalian glycans to glycoproteins at an industrial scale [30, 31]. However, costly manufacturing processes, heterogeneous products, and risk of viral contamination remain a significant challenge for production in mammalian cell lines [32–34]. As glycosylation is conserved in all eukaryotic cells, other eukaryotic expression systems such as yeast, plant and insect cells have also been employed to perform this modification [35, 36]. These systems offer the advantage of relatively faster production and lower risk of mammalian virus introduction, if non-mammalian or non-humanised glycans are acceptable within the glycoprotein product [37–39]. However, the production of protein containing a novel glycan or purely heterologous glycoprotein often requires alteration to native pathways which can potentially affect cell fitness, as many glycosylation pathways are essential for viability in eukaryotic cells [40–42].

Functional transfer of the *pgl* locus from *C. jejuni* into *E. coli*, a bacterial workhorse for recombinant protein production, opened up a new area of bacterial glycoengineering [15]. Glyco-competent *E. coli* carrying heterologous protein glycosylation machinery is an attractive platform owing to its potential as a non-virulent, rapidly growing host, with low fermentation costs [43–45]. From an engineering standpoint, the absence of a native protein glycosylation pathway in *E. coli* facilitates rational design to introduce orthogonal glycosylation pathways, without interference from endogenous GTs, hence leading to the production of homogenous products [45, 46]. In addition, one could expect a minimised effect of glycoengineering on the fitness of *E. coli* cells, as the host does not depend on protein glycosylation for cell viability. Bacterial glycoengineering also benefits from the use of the key enzyme *Cj* PglB OTase, which has been known to have relaxed substrate specificities in term of glycans and target proteins [18, 23, 24, 47]. Transfer of glycans with various size and composition has been demonstrated along with a wide range of protein recipients spanning from bacterial to eukaryotic origin. The only caveat is that the glycans require the presence of an acetamido group in the reducing-end sugar [47], and the target protein needs to contain the sequon in a structurally exposed and flexible region [18, 26]. To date, glyco-competent *E. coli* has been extensively developed in the manufacture of novel recombinant bacterial vaccines and glycoconjugates [46, 48–52]. Further, promising progress has been made to engineer glyco-competent *E. coli* to produce authentic mammalian glycans and glycoproteins [53–56], and to explore and engineer bacterial OTase with a greater substrate specificity [57–59].

Nevertheless, a common challenge of recombinant N-linked glycoprotein production in *E. coli* is inefficient glycosylation [26, 44, 45, 60]. To overcome this challenge some strains were improved by eliminating competing pathways either in glycan biosynthesis or glycan destination [61–65]. Other efforts focused on the genomic integration and expression of *pglB* and other glycosyltransferases, led to ~ twofold enhancement in glycosylation efficiency [65–67]. Glycosylation efficiency was also increased by both metabolic pathway engineering (1.2- to 1.8-fold) and process optimisation (1.3- to 1.5-fold) [33, 43, 68, 69]. Overall, glycosylation efficiency is reported to largely depend upon sequon accessibility, and so it is essential to understand how the OTase and target proteins interact during co- and post-translocational glycosylation [23–26]. In vitro studies have shown that folded protein can be glycosylated more efficiently by PglB (~ 15-fold) if the sequon is located on an exposed, flexible and unstructured region [26]. Sequon accessibility can also be enhanced in vitro by interfering with protein folding or destabilisation [23]. In contrast, coupling of glycosylation and the Sec-translocation pathway in *E. coli* improved AcrA and PEB3 glycosylation (two to fourfold) [26]. However, glycosylation efficiency of these recombinant targets remained much lower in *E. coli* compared to *C. jejuni* (up to 15-fold). Under certain fermentation conditions previously evaluated, increased glycosylation efficiency occurs in parallel with a decrease in protein production, rather than an increase in total amount of glycoprotein per cell (yield) [48]. Further, these conditions can also lead to growth defects, negatively affecting total glycoprotein per culture volume (titre) [48]. So, in order to develop alternative genetic and process strategies it is important to evaluate glycoprotein yield and titre as well as glycosylation efficiency.

Mammalian STT3A interacts directly with Sec61 translocon complex, thus increasing the local concentration of OTase-target protein substrate and allowing rapid recognition of the sequon in relaxed-unfolded regions of the protein during translocation before the protein diffuses away from the membrane [22]. Moreover, recent studies demonstrated the presence of an oxidoreductase-like protein in the eukaryotic OTase complex—Ost3p/Ost6p in yeast or N33/Tusc3 in human STT3A and B containing OTases. These oxidoreductase-like proteins modulate folding of disulphide-containing proteins in the ER lumen prior to glycosylation leading to increased sequon accessibility [70, 71]. However, to date the involvement of folding modulators has not been reported in the bacterial *N*-glycoprotein pathway.

Here, using *E. coli* as a host, we explored several strategies to maximise N-linked glycosylation including modulation of expression, sequon folding state and accessibility of the target protein, in order to increase interaction with PglB (Fig. 1C, D). The target proteins selected varied in their structure, disulphide-bond content and sequon position in order to explore the impact of different protein structural contexts upon the strategies employed. Our initial study sought to explore the influence of modulating target production level upon glycosylation efficiency. We next investigated if increasing membrane residency time of the target protein, thereby increasing local interaction between OTase PglB and the target protein could enhance glycosylation (Fig. 1C). To further explore the effect of protein folding modulation on glycosylation, we used model disulphide-bond containing proteins (Fig. 1D). These were produced under sub-optimal conditions for disulphide formation, such as under oxygen-depleted condition or in the absence of oxidoreductase (Δ*dsbB* or Δ*dsbC*), in order to mimic the ER lumen of eukaryotes. As production of recombinant protein under sub-optimal conditions can result in reduced cell viability and total protein production, we explored supplementation with the chemical oxidant cystine to recover disulphide bond-containing protein production yields in the oxidoreductase mutant (Δ*dsbB*). The various genetic and process conditions presented here demonstrated enhanced glycosylation efficiency, yield and titre, informing potential strategies to improve N-linked glycosylation in *E. coli* for the production of various disulphide containing and non-disulphide target glycoproteins.

## Results

### Selection of model proteins to assess glycosylation efficiency

**Fig. 2:**
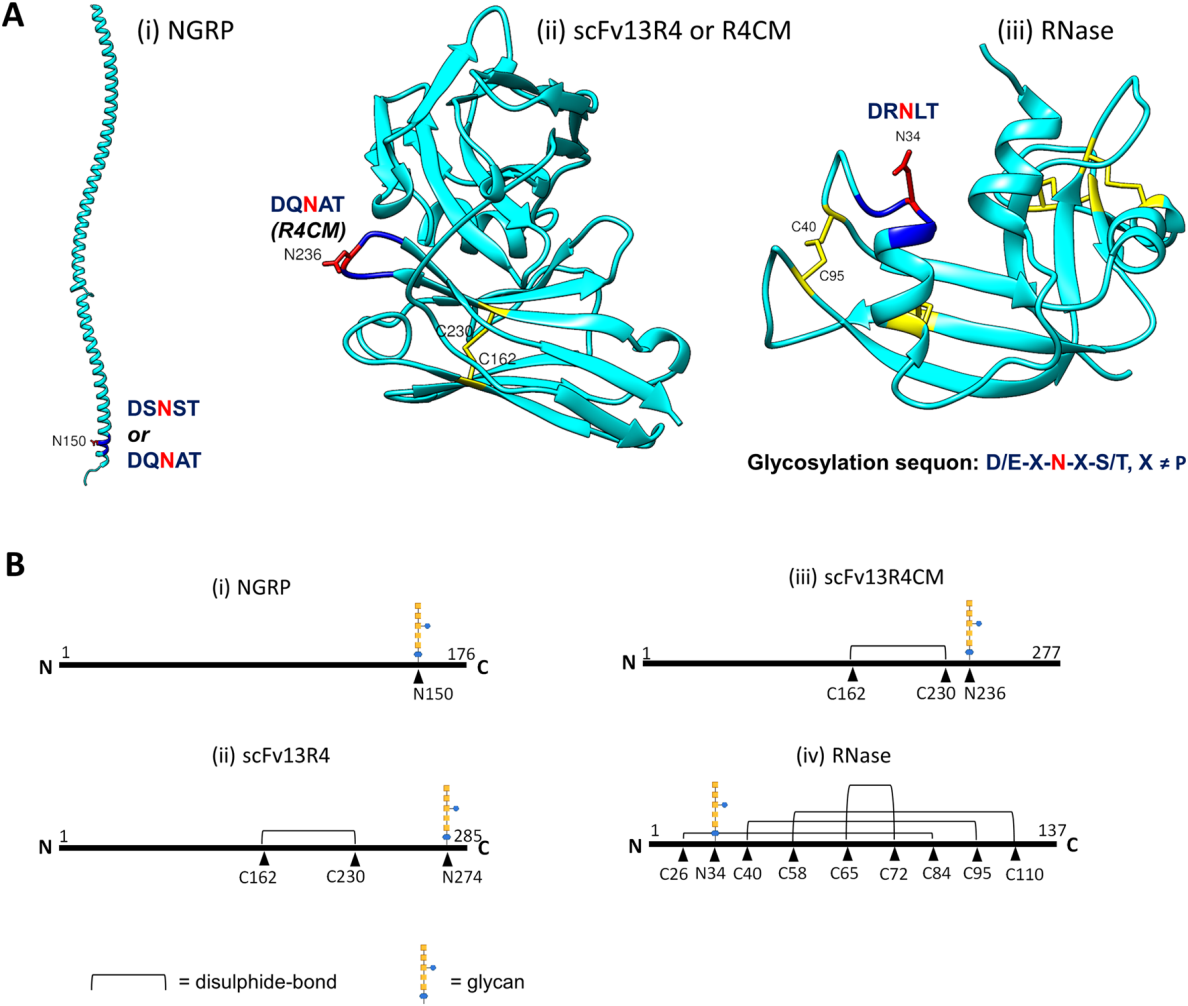
Structural variation of model glycoproteins. **A** Structural models of (i) NGRP, (ii) scFv13R4 and scFv13R4CM were generated by Phyre2 based on protein homology prediction (≥ 99% confidence) [113]. (iii) X-ray crystallographic structure of RNase A (PDB code 3WMR). Ribbon model of the proteins was drawn by UCSF Chimera [114]. Position of the sequon variants (D/E-X-N-X-S/T, X ≠ P) within the protein is indicated. Disulphide-bonds are highlighted in yellow. C-terminal sequon (DQNAT) of scFv13R4 is not displayed in the protein model. **B** Linear representation of proteins; (i) NGRP, (ii) scFv13R4, (iii) scFv13R4CM, and (iv) RNase A. Position of disulphide-bonds (C–C) and glycosylation sites (N) are indicated with amino acid positions

The four model proteins used in this study, *N*-glycosylation reporter protein NGRP, anti-β-galactosidase single-chain Fv scFv13R4 and R4CM, and bovine pancreatic ribonuclease RNase A were selected based on their diversity of protein structure, disulphide-bond content, and sequon location within the protein (Fig. 2A, B). These model proteins have been used previously to study N-linked glycosylation in *E. coli* [23, 58, 65, 72]. For simplicity all were modified by the addition of a single bacterial N-linked glycosylation sequon to assess glycosylation efficiency. A C-terminal hexahistidine tag was also added for detection of glycosylated and non-glycosylated forms by immunoblotting (anti-His). NGRP is a truncated version of the native periplasmic *C. jejuni* Cj0114 protein [73] designed to improve the performance of glycopeptide structural characterisation by mass spectrometry [58]. NGRP contains either a sequon within the C-terminal region and lacks disulphide-bonds (Fig. 2A, B, i). The scFv13R4 protein was modified by addition of a C-terminal DQNAT glycosylation sequon (Fig. 2B, ii). This sequon was repositioned closer to the cysteine residue involved in disulphide-bond formation to generate the R4CM variant (Fig. 2A ii, B iii). RNase A was modified to replace S32 with D converting the nearby native eukaryotic sequon (NLT) to the bacterial consensus sequon (DRNLT) [23]. This sequon is also adjacent to a cysteine residue involved in disulphide-bond formation (Fig. 2A iii, B iv). Varying the location of this sequon has been shown to influence glycosylation [23, 72]. scFv13R4 and R4CM are predicted to have a single disulphide-bond [74] whilst RNase A contains four, three of which are non-consecutive.

### Expression attenuation of the *NGRP* model target has limited impact upon glycosylation efficiency

**Fig. 3:**
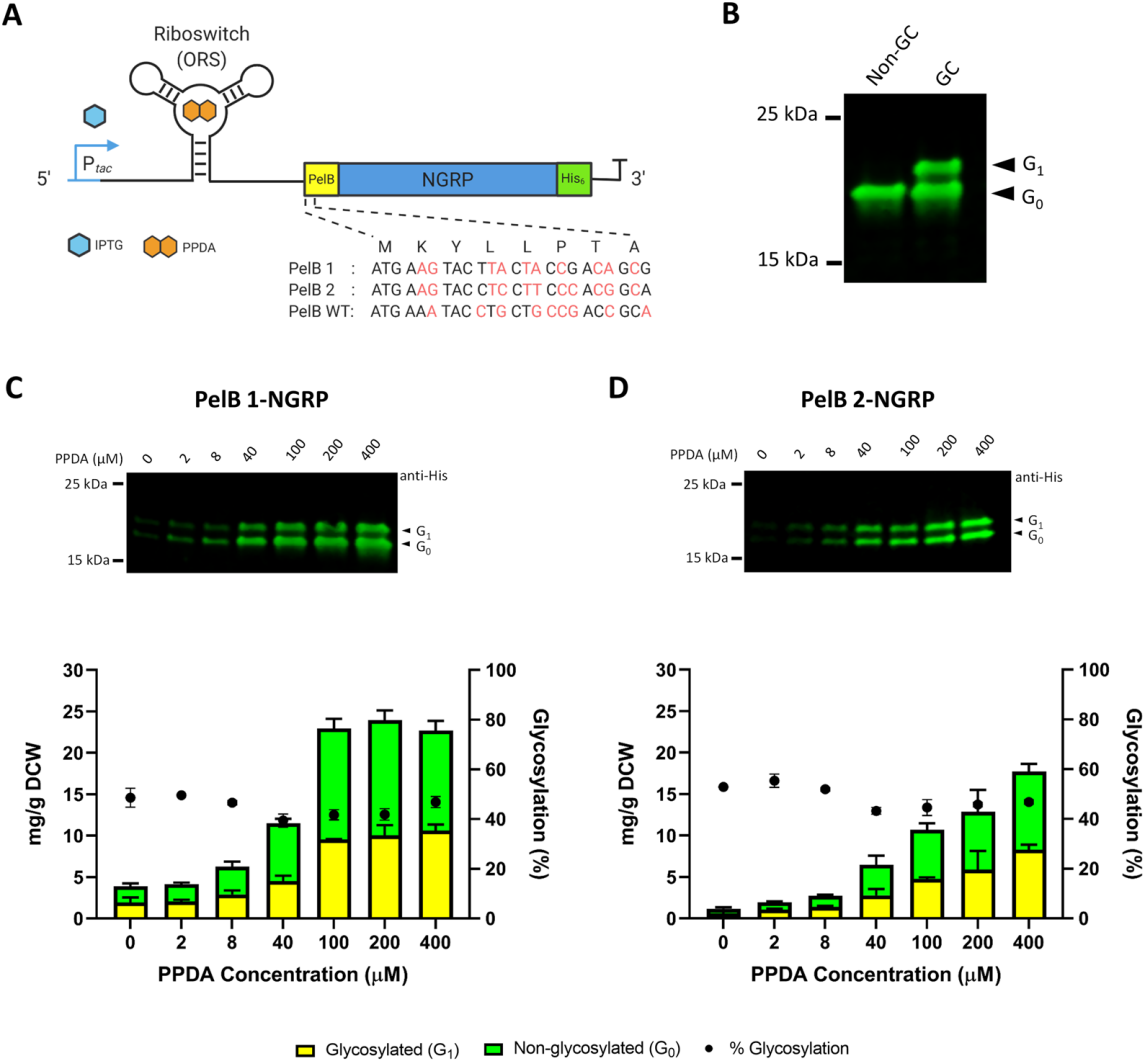
Glycosylation of NGRP in glyco-competent *E. coli*. **A** Organisation of pDEST-ORS expression vector used in this study. Target gene (NGRP) was fused with Sec signal peptide PelB (N-terminal) and Hexahistidine-tag (C-terminal). Expression of the target gene was regulated transcriptionally by P_*tac*_ via IPTG induction and translationally by orthogonal riboswitch (ORS) via PPDA titration. IPTG = Isopropyl β-d-1-thiogalactopyranoside, PPDA = Pyrimido [4,5-d] pyrimidine-2,4-diamine. Three synonymous nucleotide sequence variants of PelB-NGRP N-terminal codon (PelB 1, PelB 2, and PelB WT or wild-type) are shown. These variants were tested to explore the impact of the 5ʹ codon context upon the riboswitch-dependent regulatory function. **B** Western blot analysis of periplasmic fractions of glyco-competent (GC) and non-glycocompetent (Non-GC) strains of *E. coli*. Anti-His antibody was used to detect the NGRP. Arrows indicate non-glycosylated (G_0_) and glycosylated (G_1_) NGRP. **C**, **D** Quantitative Western blot analysis (Densitometry) of NGRP located in the periplasmic fractions from the two signal peptide variants, **C** PelB 1 and **D** PelB 2-NGRP. Proteins were produced with increasing PPDA inducer concentrations (100 μM IPTG and 0, 2, 8, 40, 100, 200, 400 μM PPDA). Total proteins were quantified using a pre-determined purified NGRP standard curve (15 ng to 100 ng) (“Methods”). The data were converted into mg/g of dry cell weight (DCW) based on normalisation and calculation with measured OD_600_ of the samples. Glycosylated (yellow bar) and non-glycosylated (green bar) protein are as shown (left y-axis). % Glycosylation (% G_1_/G_0_ + G_1_) is indicated (black circle, right y-axis). Data were processed from three biological replicates; error bars represent standard deviation from mean values. Representative Western blots are shown as insets

It was previously shown that NGRP was produced in *E. coli* as a soluble periplasmic protein [58]. Initially in this study, we aimed to explore the impact of *NGRP* expression level upon glycosylation efficiency using inducible expression vector pDEST-ORS (Fig. 3A). In this vector, expression of the target gene is regulated both transcriptionally by the IPTG-inducible *tac* promoter (P_*tac*_) and translationally by the PPDA-inducible orthogonal riboswitch (ORS) located within the 5ʹ UTR [75, 76]. The SecB-dependent signal peptide of PelB was integrated at the N-terminus of the target protein to direct secretion into the periplasm. The 5ʹ coding sequence context of genes has previously been shown to influence riboswitch conformation and gene expression levels [77]. Here, three variants containing different synonymous codons at the first eight amino acids within the PelB leader sequence were used, termed wild type (WT), PelB 1 and PelB 2-NGRP (Fig. 3A). pDEST-ORS-NGRP was transformed into *E. coli* Top10F’ expressing the *C. jejuni N*-glycosylation machinery from pACYC*pgl* (glyco-competent). Cells were cultivated in triplicate and expression induced over a range of different inducer concentrations (100 μM IPTG and 0, 2, 8, 40, 100, 200 and 400 μM PPDA) (“Methods” section). To analyse glycoprotein production, proteins were collected from periplasmic fractions. Total target protein was quantified by Western blot densitometry in which pre-determined purified protein (between 15 to 100 ng) was used as a standard for quantification (“Methods” section). The quantification data were plotted as the yield mg/g of dry cell weight (DCW) following the normalisation and conversion of measured OD_600_ into DCW (“Methods” section). Glycosylation efficiency (%) was determined from the proportion of glycosylated to total NGRP (G_1_/G_1_ + G_0_). NGRP expression in glyco-competent *E. coli* produced a single band indicative of glycosylated NGRP (~ 22 kDa) with slower migration in the gel compared to non-glycosylated NGRP (~ 20 kDa) (Fig. 3B). Previous study has confirmed by mass spectrometry that this altered mobility of NGRP is due to N-linked glycosylation [58].

For all constructs no NGRP production was detected in the absence of inducer confirming tight control of basal expression (Additional file 1: Figure S1A). Production of NGRP containing PelB-WT showed poor titratability upon induction (Additional file 1: Figure S1B–D) but titration of NGRP production was observed with increasing PPDA inducer in PelB 1 and PelB 2-NGRP constructs giving a dynamic range of expression of 6 and 15-fold respectively (expression level at Max/Min inducer concentration) (Fig. 3C, D). Interestingly, NGRP was glycosylated with similar efficiency (PelB 1-NGRP = 45 ± 4%, PelB 2-NGRP = 49 ± 5%) irrespective of the total amount of NGRP produced (PelB 1-NGRP = 3.9 ± 0.9 to 22.7 ± 1.5 mg/g DCW, PelB 2-NGRP = 1.2 ± 0.4 to 17.7 ± 1.5 mg/g DCW) (Fig. 3C, D). Correlation analysis (Pearson) showed no strong or significant relationship between glycosylation efficiency and the protein production level of NGRP (Additional file 1: Figure S2). This appears to indicate that the cell glycosylation capacity i.e., lipid-glycan substrate availability and glycosylation enzyme activity do not limit NGRP glycosylation, at least within the parameters of this experiment. Cell growth was analysed based on the optical density of cell culture at the end of incubation or induction. The result showed that increased NGRP production level has minimal but significant impact on cell growth (Pearson correlation PelB 1-NGRP, r = − 0.7645, *P* = 0.0453 PelB 2-NGRP, r = − 0.8042, *P* = 0.0292) (Additional file 1: Figure S3). As the PelB 1 variant of leader sequence gave highest maximum expression and improved dynamic range of gene expression, this variant then was used within the pDEST-ORS construct for the subsequent experiments.

### Design of a signal peptide cleavage site mutant to increase membrane residency time of target protein during glycosylation

As the gene titration analysis of NGRP indicated that glycosylation efficiency was independent of the amount of protein produced, we sought to explore if glycosylation efficiency was dependent upon the target protein residency time within the membrane, and therefore whether co-translocational glycosylation could be enhanced. To do this we constructed a system to allow membrane trapping of the target protein through modification of the PelB signal peptide cleavage site. We reasoned that by decreasing the recognition and processivity of the cleavage site by signal peptidase I (SPaseI) (Additional file 1: Figure S4), that the target protein would be trapped in the membrane leading to increased residency time between the OTase and the target protein-Sec complex. In addition to NGRP, we also used scFv13R4 as a target protein, to explore how the strategy would work with structurally distinct proteins.

The SPaseI-dependent cleavage site is located at the C terminal end of the signal peptide after the recognition motif Ala-X-Ala (X any amino acids) (Additional file 1: Figure S4) [78]. Substituting Ala with Thr at -1 from the cutting site has been shown to reduce processing [78, 79]. Another key amino acid is a proline residue around − 4 to − 6, whose structural turn promotes signal peptide alignment with SPaseI, and deletion or replacement is reported to inhibit signal peptide cleavage [79, 80]. In silico analysis (SignalP 4.1) of the resultant modified PelB signal peptide Leu-Thr-Met-Thr (TMT) motif showed a predicted 40–60% decrease in cleavage site processing compared to the wild type Pro-Ala-Met-Ala (wt) motif (Additional file 1: Figure S5A-B and C-D, table for Y-score) [81]. With only residues around the cleavage site modified in the TMT variant, 90% of Sec signal sequence was left unchanged (N, H and C-region) [82], retaining the signal for protein sorting and delivery to the translocon (Additional file 1: Figures S4, S5). PCR site-directed mutagenesis then was performed to generate a TMT mutant of PelB NGRP and scFv13R4 in the pDEST-ORS construct (“Methods” section).

**Fig. 4:**
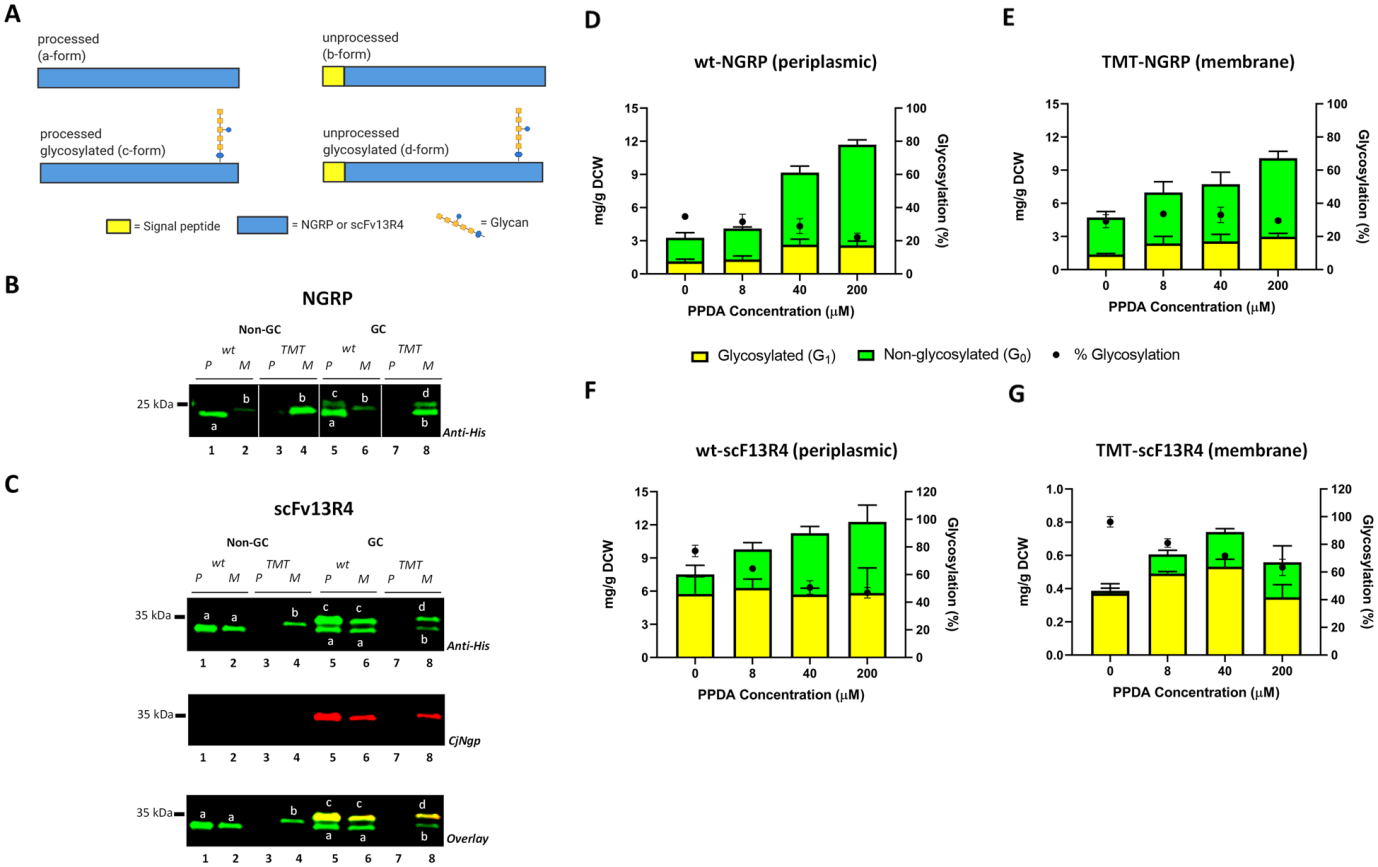
Production of NGRP and scFv13R4 isoforms containing signal peptide cleavage site variants. **A** Schematic representation of four predicted isoforms of NGRP or scFv13R4 (a-d-forms) based on their signal peptide processivity and protein glycosylation. **B**, **C** Western blot analysis of membrane (M) and periplasmic (P) expression of wild type (wt) and signal peptide cleavage mutant (TMT) NGRP (**B**) and scFv13R4 (**C**) in glyco-competent (GC) and non glyco-competent (Non-GC) *E. coli*. **B**, **C** All NGRP and scFv13R4 isoforms were detected by anti-His antibody. Glycosylated scFv13R4 was detected by CjNgp antibodies. Predicted a-d isoforms within the bands are indicated. The lanes in panel B are from the same blot while white vertical lines indicates a non-adjacent lanes. The protein migration band relative to each other in different lanes in the blot is unchanged. Additional file 1: Figure S7 shows the uncropped Western blot image of **B**. **D**–**G** Quantitative Western blot analysis (Densitometry) of membrane (TMT) and periplasmic (wt) localised **D**–**E** NGRP and **F**–**G** scFv13R4 produced in glyco-competent *E. coli*. Proteins were produced under different induction conditions (100 μM IPTG and 0, 8, 40 and 200 μM PPDA). Anti-His antibody was used to detect the proteins (glycosylated and non-glycosylated). Total proteins were quantified using a pre-determined purified NGRP or scFv13R4 standard curve (5 ng to 100 ng) (“Methods”). The data were converted into mg/g of dry cell weight (DCW) based on normalisation and calculation with measured OD_600_ of the samples. Glycosylated (yellow bar) and non-glycosylated (green bar) protein as shown (left y-axis). % Glycosylation (% G_1_/G_0_ + G_1_) is indicated (black circle, right y-axis). All data (**D**–**G**) were processed from three biological replicates. Error bars indicate standard deviation from mean values

To investigate the effect of the cleavage site mutations upon protein localisation and glycosylation, NGRP and scFv13R4 wt and TMT variants were expressed in glyco-competent (GC) and non glyco-competent (Non-GC, control) *E. coli* strains (“Methods” section). We hypothesised there would be four possible NGRP or scFv13R4 isoforms generated based on their signal peptide processivity and glycosylation state (Fig. 4A). These predicted forms were processed (a-form), unprocessed (b-form), processed glycosylated (c-form), and unprocessed glycosylated (d-form). Glycosylated and unprocessed protein will migrate slower than non-glycosylated and processed protein due to additional glycan (single, ~ 1.5 kDa) and un-cleaved signal peptide (~ 2 kDa). As expected, the fastest migrating species were observed in the periplasm, (Fig. 4B, C, *lanes 1* and *5*), and are predicted to be correctly processed (a-form). In contrast, the mutated signal peptide cleavage site clearly impacts upon the release of target protein into the periplasm fraction (Fig. 4B, C, lanes 3 and 7). Further, proteins with the TMT mutation were only observed located in the membrane fraction (Fig. 4B, C, lanes 4 and 8), which are most likely unprocessed (b-form). Glycosylated isoforms were observed as an additional higher molecular weight band in the periplasm or membrane of glyco-competent cell (predicted c or d-form) (Fig. 4B, lanes 5 and 8, and Fig. 4C, lanes 5, 6 and 8). Glycosylation was confirmed using a polyclonal CjNgp antiserum [83] but as it detects both glycosylated and non-glycosylated NGRP (Additional file 1: Figure S6) it could not be used to probe the degree of glycosylation in NGRP. However, the glycosylation status of scFv13R4 could be clearly observed (Fig. 4C). We noticed that the gel migration of some predicted isoforms could not be clearly resolved or with apparent different migration across the different lanes of the gel, (e.g., Fig. 4B, lanes 5, 6 and 8, and Fig. 4C, lanes 5 and 8). To rule out that band separation was due to differences in migration in different gel lanes, we mixed the samples (lane 5 and 8) and ran them together for an extended electrophoresis running time. By this re-running, all 4 isoforms (a–d forms) were detected as expected and the difference in migration rate was evident (Additional file 1: Figure S8). No glycosylated isoforms were observed in the non glyco-competent controls (Fig. 4B, C, lanes 1–4). Although analysis of the membrane fraction from both glyco-competent and noncompetent cells producing scFv13R4 with the wt signal peptide cleavage site produced bands (Fig. 4C, lanes 2 & 6) with similar migration as those in the periplasm fractions (a-form) (Fig. 4C, lanes 1 and 5). However, we suspect this band is correctly processed but insoluble scFv13R4 from the periplasm that has been associated with the membrane during the fractionation process, indeed this observation has been previously reported for this protein [76].

In agreement with previous findings, the ability of PglB to perform co-translocational glycosylation was supported here by observation of glycosylated pre-protein TMT-NGRP and TMT-scFv13R4 (d-form) in the membrane of glyco-competent cells (Fig. 4B, C, lanes 8) [26]. Whereas wt-NGRP seems to be glycosylated post-translocation or just before the protein release into the periplasm after signal peptide processing, since no glycosylation of the membrane bound wt-NGRP pre-protein (d-form) was detected (Fig. 4B, lane 6). Glycosylation of membrane bound NGRP was only observed for the TMT variant where an additional band in the membrane was shown, suggesting that glycan is mostly acquired during an extended residency time within the membrane (Fig. 4B, lane 8). In subsequent experiments, we performed quantitative analysis of glycosylation efficiency for the target proteins in both the membrane and in the periplasm.

### Glycosylation of NGRP and scFv13R4 during extended membrane residency time

Our data demonstrated that target proteins containing the modified cleavage site (TMT) were not released to the periplasm and were retained in the membrane. To evaluate how secretion attenuation contributed to glycosylation, the wt and TMT variants of NGRP and scFv13R4 were produced in glyco-competent *E. coli*, under different inducer concentrations (100 μM IPTG and 0, 8, 40, 200 μM PPDA) (“Methods” section). Figure 4D–G shows the quantitative Western blot analysis of protein detected in the periplasm and membrane fractions for the wt and TMT variants. We observed that total target protein production increased upon induction, but with only modest dynamic ranges observed for scFv13R4 (wt = 1.6-fold, from 7.5 ± 2.3 to 12.3 ± 3.8 mg/g DCW; TMT = 1.4-fold, from 0.39 ± 0.07 to 0.56 ± 0.17 mg/g DCW) in contrast to NGRP which displayed greater dynamic ranges (wt = 4.3-fold, from 3.3 ± 0.7 to 11.7 ± 0.6 mg/g DCW; TMT = 2.4-fold, from 4.7 ± 0.5 to 10.1 ± 0.9 mg/g DCW). Both membrane (TMT) and periplasmic (wt) fractions of NGRP yielded similar amounts of target protein under the same induction condition (Fig. 4D, E; Additional file 1: Figure S9*A*). In contrast, the membrane fraction for TMT-scFv13R4 had significantly lower target protein than the periplasmic fraction wt-scFv13R4 (~ 15- to 20-fold reduction) (Fig. 4F, G; Additional file 1: Figure S9B). One possible explanation is that the two exemplar target proteins display different intrinsic stability and proteolytic susceptibility within the membrane, which consequentially reduces the relative protein yield in the membrane compared to that observed in the periplasm fraction. As previously observed (Fig. 3C, D), the proportion of glycosylated NGRP was minimally impacted by the protein production levels, both in the periplasm fraction (wt) and in the membrane fraction (TMT) (average glycosylation efficiency wt = 29 ± 6%, TMT = 31 ± 3%) (Fig. 4D, E). Indeed, correlation analysis (Pearson) showed no significant relationship between protein level and glycosylation efficiency for wt and TMT-NGRP (Additional file 1: Figure S10A and B). Increased NGRP production levels had no negative impact upon cell growth at lower induction condition (0–40 μM PPDA) (OD_600_ wt ~ 8, TMT ~ 11) (Additional file 1: Figure S11A, B). Only at the highest induction levels (200 μM PPDA), was a reduction in cell growth observed (OD_600_ wt ~ 5, TMT ~ 3) (Additional file 1: Figure S11A, B). Overall, we did observe cell culture dependent effects, as lower NGRP glycosylation efficiency and protein yields were achieved in this experiment (Fig. 4) compared to the previous result (Fig. 3) (glycosylation efficiency mean 45 ± 4% vs 29 ± 6%, and total yield 23.94 ± 2.21 vs 11.7 ± 0.6 mg/g DCW). The previous experiment was run under microculture cultivation conditions (vs. flask) and shorter induction time (3 h vs 6 h) (see “Methods” section).

In contrast to the limited impact of titration upon glycosylation efficiency for NGRP, increased scFv13R4 production levels led to decreased glycosylation efficiency (wt = 1.6-fold, from 96 to 63%; TMT = 1.4-fold, from 77 to 47%) (Fig. 4F, G). Correlation analysis (Pearson) showed a significant inverse relationship between protein expression level and glycosylation efficiency for wt-scFv13R4 (r = − 0.9920, *P* = 0.008), while for TMT no significant correlation was observed) (Additional file 1: Figure S10C, D). Cell growth analysis showed that production of both the scFv13R4 variants (wt and TMT) led to reduced cell growth at higher protein production levels (wt OD_600_ 5.4 ± 0.2 to 2.4 ± 0.6; TMT OD_600_ 3.0 ± 0.5 to 2.1 ± 0.2) (Additional file 1: Figure S11C, D). Therefore, in addition to the effect of protein production level, reduced cell fitness could also possibly contribute to the decreasing glycosylation activity in the cell [48].

Interestingly, glycosylation efficiency of the membrane-bound TMT-scFv13R4 was always consistently higher than for the periplasmic wt-scFV13R4 under the same induction conditions (96–63% vs 77–47%, 0.015 ≤ *P* ≤ 0.08) (Fig. 4, *F* and *G*, and Additional file 1: Figure S12*A*). Whereas the glycosylation efficiency observed for the NGRP variants was similar under the same induction conditions (0–40 μM PPDA, wt 34%–28% vs. TMT 34%–29%). The exception to this observed trend was under the highest induction conditions (200 μM PPDA) which showed lower glycosylation efficiency of wt-NGRP (22%) compared to TMT-NGRP (29%) (Fig. 4D, E, and Additional file 1: Figure S12B). The lower production level of TMT-scFv13R4, when compared to the wt-scFv13R4, is a potential contributing factor to the observed increase glycosylation efficiency. However, analysis of the relationship between protein yield and glycosylation efficiency for wt-scFv13R4 by linear regression (Additional file 1: Figure S13), indicates that at lower protein production levels (≤ 4.15 mg/g DCW) the target protein would be predicted to be fully glycosylated. Therefore, it would be expected that TMT-scFv13R4 would be fully glycosylated at the yields observed in the membrane (from 0.39 ± 0.07 to 0.56 ± 0.17 mg/g DCW). Therefore, protein production levels alone are likely not the only factor that can affect TMT-scFv13R4 glycosylation, and other factors, such as sequon accessibility during membrane retention could limit glycosylation. From this analysis it is clear that protein glycosylation is a combination of glycosylation both during protein translocation through the membrane (co-translocation) and after translocation in the periplasm (post-translocation). So, to achieve maximal glycosylation efficiency optimal conditions for both processes are required. Therefore, the extended membrane residency time of the TMT variant compared to the wt, appears to be a likely contributing factor upon the observed increase in glycosylation.

Increased glycosylation efficiency of membrane-bound scFv13R4-TMT indicated that extended membrane residency time may enhance protein glycosylation compared to the when protein is released into the periplasm (scFv13R4-wt). However, since glycosylation efficiency of NGRP was not affected by enhanced membrane retention, we suspected that the structural context of the sequon within NGRP and scFv13R4 influences their respective abilities to accept glycan from PglB. Unlike NGRP, scFv13R4 contains disulphide-bonds, formation of which are important for correct protein folding and which might be impeded by membrane retention [84, 85]. Indeed, the different effects of membrane retention upon protein folding and stability of NGRP and scFv13R4 might also influence the different protein yields observed between periplasmic (wt) and membrane production (TMT) of both proteins (Fig. 4D–G). Additionally, having more complex secondary and tertiary structure than NGRP, scFv13R4 might require longer time to fold which consequently may extend the unfolded glycosylation-competent state in the membrane [85]. If formation of structural elements (e.g., disulphide-bonds) in the target protein could be impeded to modulate protein folding and glycosylation, we hypothesised that perhaps specific conditions that impede structure formation could also be exploited to enhance protein glycosylation. Thus, we explored the impact of varying both genetic and process (growth) conditions that might lead to attenuation of disulphide-bond formation, to improve production of the target recombinant glycoprotein.

### Expressing disulphide-bond containing proteins under oxygen-depleted condition enhances glycosylation efficiency

Intramolecular disulphide-bonds are essential to maintain the folding and stability of many proteins [86]. In *E. coli* and many Gram-negative bacteria, their formation is catalysed enzymatically by the dedicated disulphide-bond forming (DSB) pathway, primarily under aerobic conditions [87–90]. The key enzymes in this pathway are the periplasmic thioredoxin-like protein DsbA and the transmembrane recycling factor protein DsbB. DsbA oxidises protein substrates to give rise to a disulphide-bond, whilst reduced DsbA is quickly recycled to its active oxidised form by DsbB. DsbB is then re-oxidised by ubiquinone via the electron transport chain, in which the final electron acceptor is oxygen during aerobic growth [90]. However, during growth in low oxygen or anaerobic conditions, menaquinone mediates the transfer of electrons to alternative acceptors such as nitrate or small organic molecules such as fumarate. This leads to a reduced oxidation rate for both DsbAB relative to the aerobic pathway [87, 88].

Here we varied oxygen availability to modulate disulphide-bond formation and protein folding and monitored consequent protein glycosylation levels. To vary the oxygen transfer to cells in liquid media, cultures were grown in shake flasks with different culture to flask volume ratios (5:50, 10:50, and 25:50 ml) (“Methods” section). As described scFv13R4, scFv13R4CM, and also RNase A were modified with glycosylation sequons and used as the target disulphide-bond containing proteins and NGRP (DQNAT) acted as a non-disulphide bond containing control protein.

**Fig. 5:**
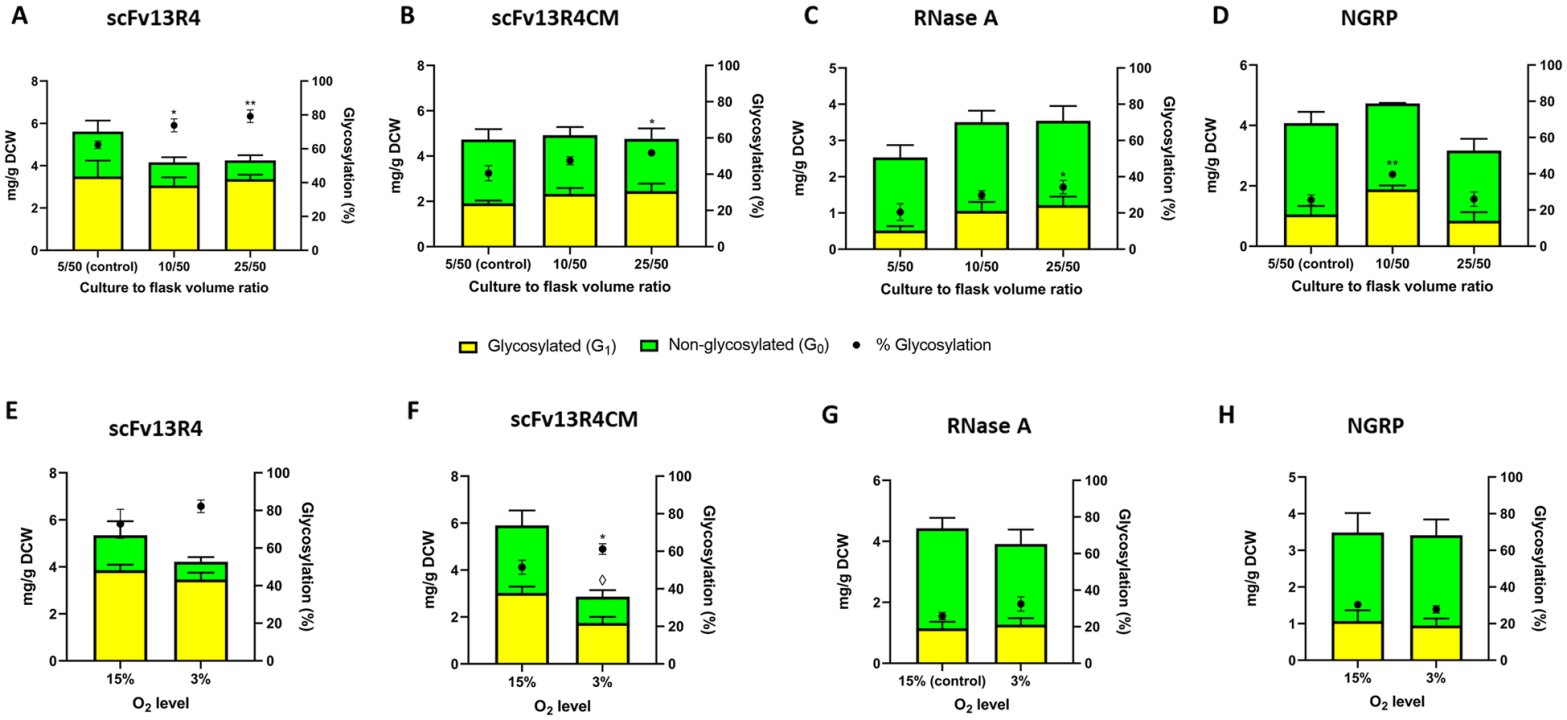
Effect of culture conditions and oxygen availability upon target protein glycosylation in *E. coli*. **A**–**D** Effect of culture to flask volume ratio (oxygen transfer efficiency) upon target protein glycosylation in *E. coli*. Quantitative Western blot analysis (densitometry) of **A** scFv13R4, **B** scFv13R4CM, **C** RNase A and **D** non-disulphide control protein NGRP located in periplasm of glyco-competent *E. coli* in shake flask under three different cultures to flask volume ratio (5:50, 10:50, and 25:50 ml). **E**–**H** Quantitative Western blot analysis (densitometry) of **E** scFv13R4, **F** scFv13R4CM, **G** RNase A and **H** NGRP control non-disulphide bond-containing protein, detected in the periplasm of glyco-competent *E. coli* under different oxygen levels culture (3% and 15% O_2_). **A**–**H** Total proteins were quantified using pre-determined purified scFv13R4CM, RNase, or NGRP standard curve (5 ng to 100 ng). The data were converted into mg/g of dry cell weight (DCW) based on normalisation and calculation with measured OD_600_ of the samples. **A**–**H** Glycosylated (yellow bar) and non-glycosylated (green bar) protein as shown (left y-axis). % Glycosylation (% G_1_/G_0_ + G_1_) is indicated (black circle, right y-axis). Statistical analysis was conducted by unpaired t-test with Welch’s correction to control sample at lowest culture to flask volume ratio 5:50 (**A**–**D**) or to control normoxic culture (**E–H**) (*P* < 0.05*, < 0.01**, for % glycosylation; *P* < 0.05^◊^, for total protein mg/g DCW). All data were processed from three biological replicates. Error bars indicate standard deviation from mean values

A modest but significant increase in glycosylation efficiency was observed when disulphide-bond containing proteins were produced in higher culture to flask volume ratio (5:50 to 25:50 ml; scFv13R4 62 to 79%, *P* = 0.005; scFv13R4CM 41 to 52%, *P* = 0.028; RNase A 20 to 34%, *P* = 0.017) (Fig. 5A–C). A rise in glycosylation efficiency was also examined for the control NGRP when the cells were grown at higher culture to flask volume ratio (5:50 to 10:50 ml, 25% to 40%, *P* = 0.004) (Fig. 5D). However, in contrast to the trend observed for disulphide bond-containing proteins, NGRP glycosylation efficiency decreased when the cells were grown at the highest culture to flask volume ratio (10:50 to 25:50 ml, 40% to 26%, *P* = 0.017—Fig. 5D). Each protein was produced at generally similar levels in these different conditions (mean yield scFv13R4 = 4.7 ± 1.0 mg/g DCW, scFv13R4CM = 4.8 ± 0.6 mg/g DCW, RNase A = 2.9 ± 0.6 mg/g DCW, NGRP = 4.0 ± 0.8 mg/g DCW). scFv13R4 protein production saw a small, but not significant, decrease at higher culture ratios (1.3-fold, 5:50 to 25:50), and RNase A also saw a small, but not significant, increase (1.3-fold, 5:50 to 25:50) (Fig. 5A, C). In addition, correlation analysis (Pearson) showed that the change in glycosylation efficiency was not significantly associated with the production level of disulphide-bond proteins during growth in different culture to flask volume ratio (Additional file 1: Figure S14).

In order to further explore the effect of lower oxygen culture conditions upon protein glycosylation, cells were grown in a hypoxic chamber with 3% (hypoxic) or 15% O_2_ (normoxic) (“Methods” section). Production under hypoxic conditions also increased glycosylation efficiency of disulphide bond-containing proteins, however only scFv13R4CM showed a significant change (15% to 3% O_2_; % glycosylation scFv13R4 73% to 82%, scFv13R4CM 51% to 61% *P* = 0.027, RNase A 26% to 32%, NGRP 30% to 28%), which may also be attributed to the observed greater reduction in scFv13R4CM protein yield (scFv13R4CM from 5.9 ± 0.9 to 2.9 ± 0.5 mg/g DCW, *P* = 0.005) (Fig. 5E–H). To test if glycosylation of disulphide bond-containing proteins was more sensitive to the change in oxygen levels compared to glycosylation of non-disulphide bond-containing proteins (NGRP), we quantified the fold-change of glycosylation between the proteins expressed in hypoxic (treatment) and normoxic (control) conditions. This fold-change is defined as relative glycosylation efficiency (RGE) was calculated as follows:

$${\text{RGE}} = \left( {\frac{{{\text{Glycosylation}}\;{\text{efficiency}}\;{\text{under}}\;{\text{treatment}}\;\left( {\text{G1/G1 + G0}} \right)}}{{{\text{Glycosylation}}\;{\text{efficiency}}\;{\text{of}}\;{\text{the}}\;{\text{control}}\;\left( {\text{G1/G1 + G0}} \right)}}} \right)$$

Therefore, even if glycosylation of non-disulphide bond-containing protein were to change in a similar direction as the model disulphide bond-containing protein, the degree of change could be compared using the RGE metric. The RGE results showed that disulphide bond-containing proteins experienced a modest increase in glycosylation fold-change during hypoxic treatment (scFv13R4 1.1-fold, scFv13R4CM 1.2-fold, RNase A 1.3-fold) but this was significantly different to the change in glycosylation observed for NGRP under the same conditions (0.92-fold) (*P* = 2.0 × 10^–4^, 3.3 × 10^–6^, 8.3 × 10^–5^ respectively) (Additional file 1: Figure S15).

While the glycosylation efficiency of disulphide bond-containing proteins increased when produced under oxygen-depleted conditions, cell growth was also dramatically reduced (Table 1). The negative impact upon growth seems independent of target protein and most likely related to cellular oxygen requirements, since both cells expressing disulphide or non-disulphide bond-containing proteins were negatively affected. Volumetric production (titre) of recombinant protein was decreased as a result of reduced cell growth during production in oxygen depleted conditions (Table 1).

**Table 1:** Comparative cell growth and volumetric productivity (protein titre) during recombinant disulphide-bond protein production in oxygen-depleted conditions

Proteins	Cell growth^a^ and protein titre^b^				
	Culture to flask volume ratio (Oxygen transfer)			Oxygen level	
	5:50^c^	10:50	25:50	15%^d^	3%
scFv13R4	5.49 ± 0.05	4.35 ± 0.17	2.85 ± 0.05	5.57 ± 0.09	2.43 ± 0.05
	10.78 ± 2.37 mg/L	6.30 ± 0.63 mg/L	4.25 ± 0.49 mg/L	10.40 ± 1.39 mg/L	3.59. ± 0.45 mg/L
scFv13R4CM	5.01 ± 0.12	4.13 ± 0.09	2.83 ± 0.13	4.43 ± 0.05	2.34 ± 0.05
	8.29 ± 0.76 mg/L	7.13 ± 1.01 mg/L	4.72 ± 0.88 mg/L	9.15 ± 1.39 mg/L	2.35 ± 0.40 mg/L
RNase A	3.65 ± 0.09	2.56 ± 0.00	1.84 ± 0.08	2.93 ± 0.05	1.92 ± 0.00
	3.24 ± 0.49 mg/L	3.14 ± 0.50 mg/L	2.27 ± 0.29 mg/L	4.55 ± 0.60 mg/L	2.63 ± 0.41 mg/L
NGRP	8.87 ± 0.46	5.33 ± 0.17	3.31 ± 0.09	4.83 ± 0.17	2.51 ± 0.05
	12.65 ± 2.11 mg/L	8.83 ± 0.40 mg/L	3.66 ± 0.78 mg/L	5.88 ± 1.40 mg/L	2.99 ± 0.52 mg/L

^a^Final cell density was measured as Abs_600_ (Final OD_600_) recorded 4 h after induction

^b^Total protein titre was determined from the Western blot quantification data (glycosylated and non-glycosylated), normalised and converted from sample OD_600_ of periplasmic fraction and sample volume for Western blot. For the final cell density and protein titre, the average of three biological replicates is shown and the errors indicate standard deviation

^c^5, 10, and 25 ml of cell culture were grown in 50 ml shake flask to have culture with different culture to flask volume ratio

^d^Variation in oxygen level was performed by growing the cells in 24-well plate incubated in hypoxic chamber exposed to 3% O_2_ (hypoxic) or 15% O_2_ (normoxic). The change (increasing or decreasing) of cell growth and protein titre relative to control (cell cultivation under more oxygenated conditions, 5/50 culture to flask volume ratio or 15% O_2_) was analysed by t-test with Welch’s correction (Additional file 2: Table S2 and S3)

### Production and glycosylation of disulphide bond-containing proteins in the absence of oxidoreductase DsbB

Studies have shown that in the absence of DsbA or DsbB, in order to maintain cell viability and disulphide-bond formation either additional oxygen or media supplementation with a strong oxidant is required [91]. Nevertheless, the rate of disulphide-bond formation driven directly by oxygen supplementation in these knockout strains does not fully rescue the reaction catalysed by the DSB pathway [88, 91]. The previous experiments demonstrated that producing disulphide bond-containing proteins under suboptimal conditions for disulphide-bond formation might delay protein folding, leading to enhanced interaction time with OTase and the sequon, and greater glycan transfer. Therefore, we hypothesised that when disulphide bond-containing proteins were produced in glyco-competent *E. coli* lacking one of DsbAB enzymes, such as in the Δ*dsbB* strain, glycosylation would be enhanced. To test this, a glyco-competent Δ*dsbB* strain was transformed with expression vectors encoding the model disulphide bond-containing proteins and control NGRP, and glycosylation analysed following standard shake flask cultivation (“Methods” section).

**Fig. 6:**
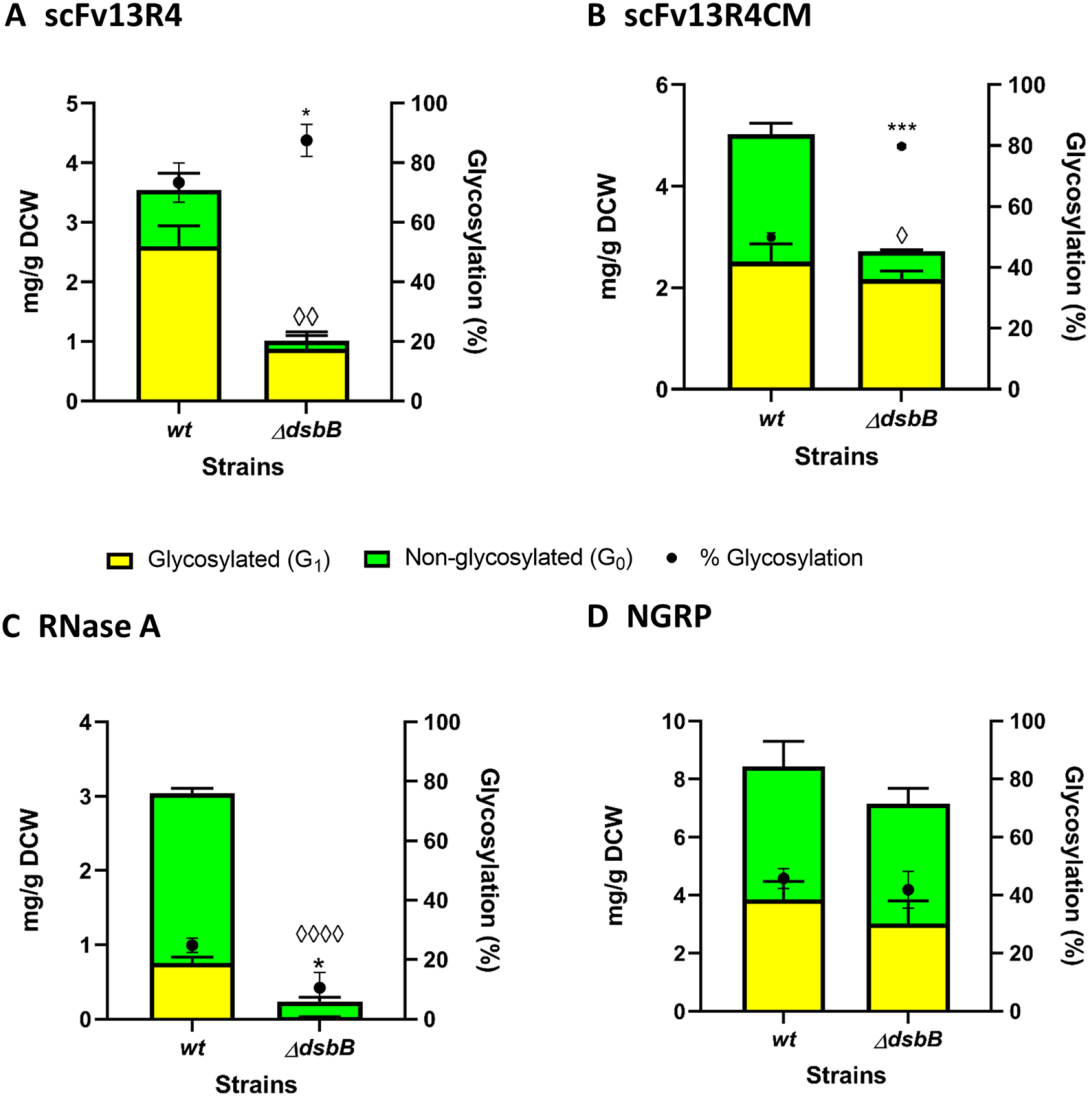
Glycosylation of disulphide bond-containing proteins in oxidoreductase mutant (Δ*dsbB*) of *E. coli*. Quantitative Western blot analysis (densitometry) of **A** scFv13R4, **B** scFv13R4CM, **C** RNase A and **D** control non-disulphide bond-containing protein NGRP expressed in periplasmic of glyco-competent *E. coli wild-type* (*wt*) or Δ*dsbB* strain. Total proteins (**A**–**D**) were quantified using pre-determined purified scFv13R4CM, RNase A, or NGRP standard curve (5 ng to 100 ng). The data were converted into yield mg/g of dry cell weight (DCW) based on normalisation and calculation with measured OD_600_ of the samples Glycosylated (yellow bar) and non-glycosylated (green bar) protein as shown (left y-axis). % Glycosylation (% G_1_/G_0_ + G_1_) is indicated (black circle, right y-axis). Additional file 1: Figure S16, *A* and *B* showed representative Western blot images for scFv13R4CM and RNase A produced in *wt* and Δ*dsbB* strains. Representative Western blot image for NGRP and scFv13R4 have been shown before in Figs. 3B, C, and 4B, C. Statistical analysis was conducted by unpaired t-test with Welch’s correction to control sample expressed in *wt* strain (*P* < 0.05*, < 0.01**, for % glycosylation; *P* < 0.05^◊^, < 0.01^◊◊^, < 0.0001^◊◊◊◊^, for total protein mg/g DCW). All data were processed from three biological replicates. Error bars indicate standard deviation from mean values

Densitometric analysis of periplasmic proteins demonstrated increased glycosylation efficiency of scFv13R4 and scFv13R4CM in the Δ*dsbB* strain compared to the *wild-type* (*wt*) strain (scFv13R4 73% to 88%, *P* = 0.047; scFv13R4CM 50% to 80%, *P* = 1.1 × 10^–4^) (Fig. 6A, B). However, expression in the Δ*dsbB* strain showed a negative effect for glycosylation of RNase A (25% to 10%, *P* = 0.025) (Fig. 6C). In parallel a significant reduction in protein production (50–90%) was observed for all three disulphide-containing model proteins in Δ*dsbB* relative to the *wt* strain (scFv13R4 from 3.5 ± 0.4 to 1.0 ± 0.3 mg/g DCW, *P* = 1.7 × 10^–3^; scFv13R4CM from 5.0 ± 0.6 to 2.7 ± 0.2 mg/g DCW, *P* = 1.4 × 10^–2^; RNase A from 3.0 ± 0.1 to 0.24 ± 0.05 mg/g DCW, *P* = 5.9 × 10^–7^). We suspected that the proteins might be prone to degradation following an extended delay in disulphide-bond formation and protein folding in the absence of the DsbB enzyme [89, 90]. Indeed, consistent with this notion, RNase A production was most severely affected since it has four disulphide-bonds compared to one for scFv13R4 and scFv13R4CM (Fig. 2A ii–iii and B ii–iv). However, as glycosylation efficiency can be impacted by lower amounts of target protein substrate, increased glycosylation efficiency of scFv13R4 and scFv13R4CM cannot be attributed solely to the delay of disulphide-bond formation during expression in the Δ*dsbB* strain. No significant change in total protein and glycosylation efficiency of control NGRP was observed in the Δ*dsbB* strain in comparison to the *wt* strain (*wt* = 8.5 ± 1.4 mg/g DCW, Δ*dsbB* = 7.2 ± 1.2 mg/g DCW) (Fig. 6D). The result indicates the specific effect of DsbB activity upon production and glycosylation of the target disulphide bond-containing proteins.

Table 2 summarises the comparative growth and volumetric production (recombinant protein titre) in *wild-type* and Δ*dsbB* strains producing model glycoproteins. Reduction in cell growth was observed when scFv13R4 and scFv13R4CM were produced in Δ*dsbB* relative to the *wt* strain. The reduction in cell growth along with protein production per cell (protein yield mg/g DCW) results in lower scFv13R4 and scFv13R4CM protein titres (Fig. 6A, B; Table 2). In contrast, both *wild-type* and Δ*dsbB* strains expressing RNase A showed similar growth (Table 2). Therefore, the observed reduction in RNase A protein titre (Table 2) was predominately impacted by the decrease in protein per cell (yield) (Fig. 6C, D). No difference in the cell growth was observed between *wild-type* and Δ*dsbB* expressing non-disulphide bond-containing protein NGRP as well as the protein titre produced between both strains.

**Table 2:** Comparative cell growth and volumetric production (protein titre) during recombinant disulphide-bond protein production in the *wild-type* (*wt*) and Δ*dsbB* strain

Proteins	Cell growth^a^ and protein titre^b^	
	Strains	
	*Wild-type* (wt)	Δ*dsbB*
scFv13R4	5.04 ± 0.14	3.27 ± 0.08
	6.25 ± 0.73 mg/L	1.16 ± 0.42 mg/L
scFv13R4CM	5.40 ± 0.20	3.68 ± 0.00
	9.48 ± 1.00 mg/L	3.50 ± 0.23 mg/L
RNase A	2.96 ± 0.08	3.05 ± 0.05
	3.15 ± 0.14 mg/L	0.26 ± 0.06 mg/L
NGRP	4.43 ± 0.06	4.43 ± 0.11
	13.11 ± 2.25 mg/L	11.12 ± 1.93 mg/L

^a^Final cell density was measured as Abs_600_ recorded 4 h after induction

^b^Total protein titre was determined from the Western blot quantification data (glycosylated and non-glycosylated), normalised and converted from sample OD_600_ of periplasmic fraction and sample volume for Western blot. For the final cell density and protein titre, the average of three biological replicates is shown and the errors indicate standard deviation. Cell culture was performed under standard cultivation condition in shake flask 10:50 ml. The change (increasing or decreasing) of cell growth and protein titre relative to control (production in *wt* strain) was analysed by t-test with Welch’s correction (Additional file 2: Table S4)

### Supplementation with chemical oxidant (cystine) improves production of disulphide bond-containing glycoproteins in the *ΔdsbB* strain

**Fig. 7:**
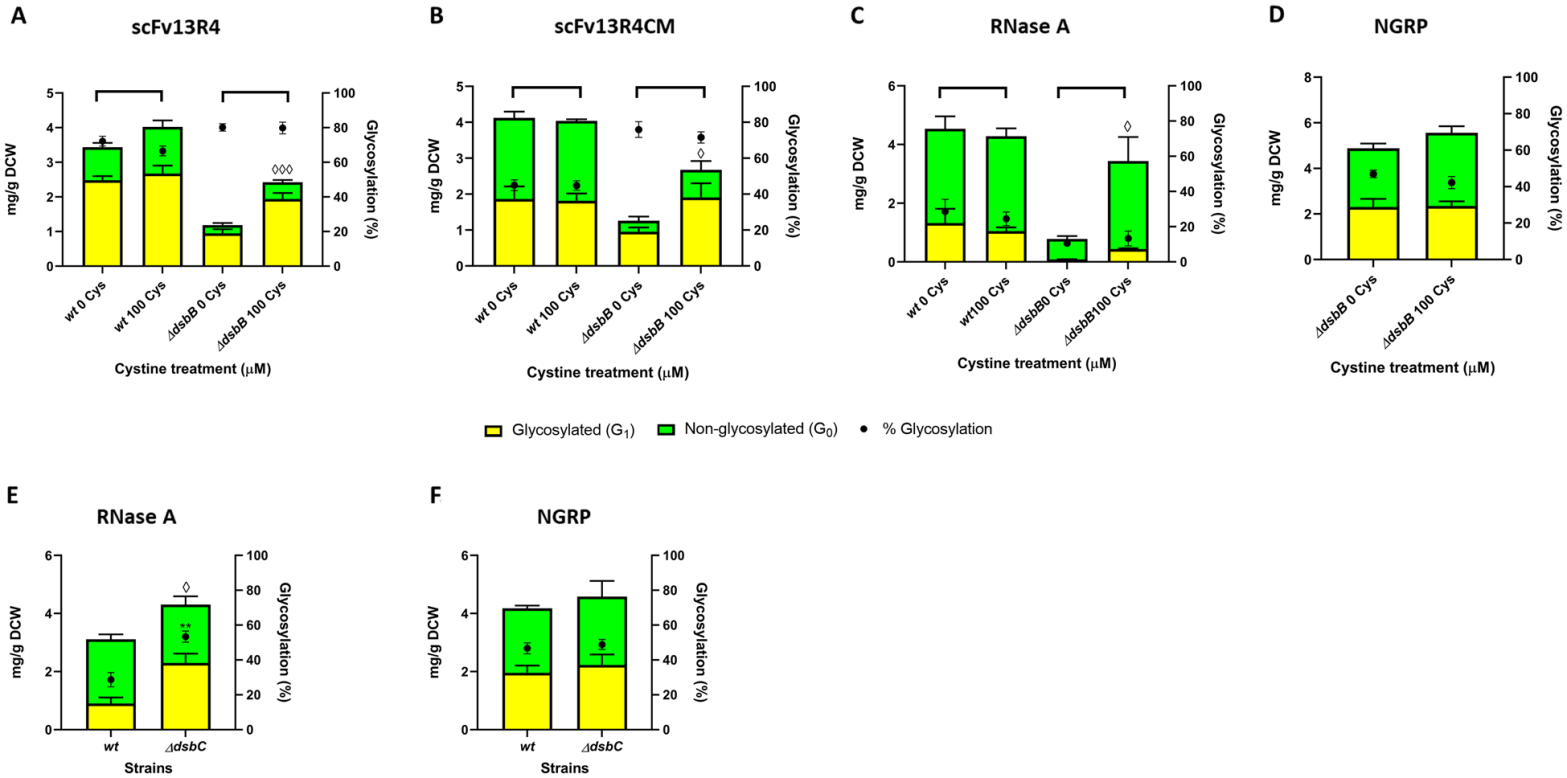
Impact of cystine supplementation upon glycosylation of recombinant proteins in Δ*dsbB* strain and glycosylation of RNase A in disulphide-bond isomerase mutant (Δ*dsbC*). (A-D) Quantitative Western blot analysis (densitometry) of **A** scFv13R4, **B** scFv13R4CM, **C** RNase A and **D** NGRP non-disulphide control protein produced in the periplasm of glyco-competent *E. coli wild-type* (*wt*) or Δ*dsbB* strain supplemented with or without 100 μM cystine during protein expression. **E**, **F** Quantitative Western blot analysis (densitometry) of **E** RNase A and **F** control non-disulphide bond-containing protein NGRP expressed in periplasmic of glyco-competent *E. coli* Δ*dsbC*. Total proteins (**A**–**F**) were quantified using pre-determined purified scFv13R4CM, RNase, or NGRP standard curve (5 ng to 100 ng). The data were converted into mg/g of dry cell weight (DCW) based on normalisation and calculation with measured OD_600_ of the samples. Glycosylated (yellow bar) and non-glycosylated (green bar) protein as shown (left y-axis). % Glycosylation (% G_1_/G_0_ + G_1_) is indicated (black circle, right y-axis). Statistical analysis was conducted by unpaired t-test with Welch’s correction to control sample expressed in *wt* or Δ*dsbB* strain without cystine treatment (**A**–**D**) or to control sample expressed in *wt* strain (**E**, **F**) (*P* < 0.05*, < 0.01**, for % glycosylation; *P* < 0.05^◊^, < 0.001^◊◊◊^, for normalised total protein). All data were processed from three biological replicates. Error bars indicate standard deviation from mean values

The above analysis shows that producing disulphide bond-containing proteins in the absence of DsbB reduces recombinant protein production (yield and titre) and cell growth. Thus, we investigated if supplementation with a small molecule oxidant (e.g. cystine) could maintain enhanced glycosylation efficiency but also recover the total target protein production levels and cell growth in the Δ*dsbB* strain. Moreover, alternative oxidants may provide different rates of modulation for disulphide-bond formation and protein folding compared to DsbAB enzymes. In this way glycosylation efficiency could be improved without heavily sacrificing protein production levels. To perform this experiment, *wild-type* (*wt*) and Δ*dsbB* strains of glyco-competent *E. coli* were prepared as before, but now 100 μM of cystine was added into the culture medium along with inducers (“Methods” section). Total production levels for all disulphide bond-containing proteins increased in the Δ*dsbB* strain upon supplementation with cystine (scFv13R4 2.0-fold, from 1.2 ± 0.2 to 2.4 ± 0.1 mg/g DCW, *P* = 9.6 × 10^–4^; scFv13R4CM 2.1-fold, from 1.3 ± 0.2 to 2.7 ± 0.6 mg/g DCW, *P* = 0.047; RNase A 5.0-fold, from 0.78 ± 0.11 to 3.4 ± 0.8 mg/g DCW, *P* = 1.4 × 10^–5^) (Fig. 7A–C). In addition, glycosylation efficiency was almost identical with and without cystine treatment even though increased protein yields were observed (average glycosylation efficiency ± cystine treatment; scFv13R4 Δ*dsbB* = 80 ± 2%, scFv13R4CM Δ*dsbB* = 74 ± 4%, RNase A Δ*dsbB* = 12 ± 3%) (Fig. 7A–C). Therefore, resulting in an increase in the absolute amounts of glycoprotein under this treatment. The scFv13R4 produced in Δ*dsbB* strain was also evaluated for the binding activity against its cognate antigen β-galactosidase (“Methods” section). The result showed increased specific binding activity of scFv13R4 from the Δ*dsbB* strain compared to that produced in the *wild-type* strain (Δ*dsbB* 0 cys = 1.8-fold, Δ*dsbB* 100 cys = 1.4-fold) (Additional file 1: Figure S17). It has been reported previously that in vitro glycosylation of scFv13R4 with *Cj* heptasaccharide does not affect protein activity [92]. However, glycosylation enhanced activity of another exemplar single chain Fv (murine 3D5 scFv) when produced in vivo (in glyco-competent *E. coli*), has been reported [72]. As such, this present analysis is consistent and indicates enhanced activity of the scFv13R4 glycoconjugate when produced via in vivo glycosylation.

Cystine function appears to be redundant in the presence of DsbAB, as there was no improvement in the production of disulphide bond-containing proteins in the *wild-type* strain (Fig. 7A–C). While RNase A production in Δ*dsbB* strain was considerably improved by cystine supplementation, glycosylation in this strain was still negatively affected relative to the *wild-type* (glycosylation efficiency *wt* = 27 ± 5%, *ΔdsbB* = 12 ± 3%) (Fig. 7C). No difference in NGRP production and glycosylation were observed when produced in Δ*dsbB* strain with or without cystine (average yield = 5.2 ± 0.5 mg/g DCW, average glycosylation efficiency = 45 ± 4%) (Fig. 7D). The result confirmed the specific effect of cystine treatment only upon disulphide bond-containing proteins.

In addition to improvement in total protein yield and absolute amounts of glycoprotein, cystine treatment also produced a better growth phenotype for the Δ*dsbB* strain producing disulphide bond-containing proteins, while the growth level was not fully restored to the level observed for the untreated *wild-type* (Table 3). Improvement in protein production per cell (yield) and cell growth resulted in increasing disulphide-bond protein titre produced in Δ*dsbB* strain under cystine treatment (Table 3). The *wild-type* strain expressing disulphide bond-containing proteins grew similarly with or without cystine supplementation (Table 3). Further, cystine addition appeared to have no impact upon the growth of the Δ*dsbB* strain producing non-disulphide bond-containing protein NGRP and the protein titre was produced similarly (Table 3). These data indicate that stable production of recombinant disulphide bond-containing proteins is responsible for the growth improvement exhibited in Δ*dsbB* strain, rather than a more global impact upon cell redox state due to the absence of DsbB.

**Table 3:** Comparative cell growth and volumetric production (protein titre) during recombinant disulphide-bond protein production in the *wild-type* (*wt*) and Δ*dsbB* strain supplemented with small molecule oxidant (cystine)

Proteins	Cell growth^a^ and protein titre			
	*Wild-type* (*wt*)		*ΔdsbB*	
	0 Cys^c^	100 Cys	0 Cys	100 Cys
scFv13R4	4.88 ± 0.08	4.91 ± 0.20	3.08 ± 0.10	3.50 ± 0.05
	5.87 ± 0.21 mg/L	6.89 ± 0.37 mg/L	1.28 ± 0.24 mg/L	2.97 ± 0.14 mg/L
scFv13R4CM	4.45 ± 0.09	4.35 ± 0.09	2.90 ± 0.00	3.65 ± 0.00
	6.42 ± 0.70 mg/L	6.15 ± 0.41 mg/L	1.28 ± 0.24 mg/L	3.42 ± 0.81 mg/L
RNase A	3.55 ± 0.05	3.60 ± 0.14	3.12 ± 0.00	3.39 ± 0.05
	5.64 ± 1.05 mg/L	5.40 ± 0.36 mg/L	0.85 ± 0.12 mg/L	4.07 ± 0.89 mg/L
NGRP	n.d	n.d	3.90 ± 0.00	3.83 ± 0.06
			6.66 ± 0.77 mg/L	7.46 ± 0.33 mg/L

^a^Final cell density was measured as Abs_600_ recorded 4 h after induction

^b^Total protein titre was determined from the Western blot quantification data (glycosylated and non-glycosylated), normalised and converted from sample OD_600_ of periplasmic fraction and sample volume for Western blot. For the final cell density and protein titre, the average of three biological replicates is shown and the errors indicate standard deviation. cell culture was grown under standard cultivation condition in shake flask 10:50 ml

^c^Culture medium was added with or without 100 μM of cystine at the same time with inducers addition. n.d. not determined. The change (increasing or decreasing) of cell growth and protein titre relative to control (production in *wt* or Δ*dsbB* strain without cystine supplementation) was analysed by t-test with Welch’s correction (Additional file 2: Table S5)

It is worthy of mention that we also noticed a difference in glycosylation efficiency improvement among disulphide bond-containing proteins during the expression in the Δ*dsbB* strain compared to the production under oxygen-depleted conditions. The increase in glycosylation efficiency of scFv13R4CM in Δ*dsbB* strain relative to *wt* strain was higher (up to 1.6-fold) (Figs. 6B and 7B) than compared to production under low oxygen transfer conditions (25/50 vs. 5/50 flask to culture vol ratio) (1.3-fold) (Fig. 5B). Both conditions gave a similar improvement in glycosylation efficiency for scFv13R4 (1.2- to 1.3-fold) (Figs. 5A, 6A, 7A). While glycosylation efficiency of RNase A was enhanced during low oxygen transfer (1.7-fold) (Fig. 5C) and hypoxic growth/low oxygen level (1.3-fold) (Fig. 5G). It is unclear why RNase A glycosylation was negatively affected during expression in Δ*dsbB*, even more considering the expression level was also greatly reduced (Figs. 6C and 7C). We speculate that without DsbAB assistance, RNase A forms an intermediate conformation which has reduced interaction with OTase. Different strategies to enhance glycosylation may be required to optimise folding-dependent glycosylation of RNase A in vivo.

### Production of RNase A in the absence of disulphide-bond isomerase (Δ*dsbC*) improves glycosylation efficiency

Cystine supplementation was shown to increase the production of RNase A in a Δ*dsbB* strain of *E. coli* (yield and titre) (Fig. 7C). However, glycosylation efficiency remained low compared with production in the *wild-type* strain. Due to the presence of non-consecutive disulphides, RNase A is more likely to be sensitive to incorrect pairing during its folding in the periplasm [90, 93]. In the periplasm, mispaired disulphide-bonds are recognised by the disulphide-bond isomerase DsbC, which catalyses reshuffling reactions of the mispaired bonds [90, 94]. Previously, Kowarik et al. demonstrated in vitro that rapid oxidation of RNase A generated mixed disulphide isoforms as preferable substrates for bacterial OTase (PglB) [23]. Another in vitro study showed the formation of intermediate disulphide isoforms were dominantly observed within RNase A in the absence of DsbC [93]. Indeed, the activity of the oxidoreductase subunit in some eukaryotic OTases leads to transient mixed disulphide forms of the target proteins, promoting an intermediate folding state, and consequently increasing their window for glycosylation [71, 95].

To investigate if RNase A glycosylation could be improved in the same way in vivo (in bacteria) by attempting to increase intermediate mixed disulphide forms, protein production was performed in a Δ*dsbC* strain of glyco-competent *E. coli*. Analysis of the periplasmic fraction showed increasing glycosylation efficiency of RNase A produced in Δ*dsbC* (29% to 53%, *P* = 0.001) (Fig. 7E). Total protein was also higher compared with production in the *wt* strain (1.4-fold, from 3.1 ± 0.3 to 4.3 ± 0.5 mg/g DCW, *P* = 0.042) (Fig. 7E). NGRP production and glycosylation was similar in both *wt* and Δ*dsbC* strain (protein = 4.4 ± 0.6 mg/g DCW, % glycosylation = 48 ± 3%) indicating no effect upon the non-disulphide bond-containing protein (Fig. 7F). Production of scFv13R4 and scFv13R4CM in the Δ*dsbC* strain resulted in compromised protein production levels (scFv13R4, 1.6-fold, from 4.8 ± 0.4 to 2.9 ± 0.3 mg/g DCW, *P* = 0.004; scFv13R4CM, 1.2-fold, from 4.8 ± 0.1 to 4.1 ± 0.4 mg/g DCW), and minor decreases in protein glycosylation efficiency (scFv13R4, from 70 to 65%; scFv13R4CM, from 49 to 39%, *P* = 0.002) (Additional file 1: Figure S18). While the isomerase activity is not necessary for proteins with only single disulphide-bonds or those possessing consecutive disulphide-bonds, it has been reported that DsbC displays a chaperone function to improve stability and solubility of various scFv model proteins [96–98].

To confirm that RNase A produced in Δ*dsbC* strain increases production of intermediate folding form of RNase, we analysed the activity of RNase produced from this strain (“Methods” section). A recent study reported that glycosylation of RNase A produced in glyco-competent *E. coli* had a similar activity with its non-glycosylated version [99]. Therefore, any decrease in the activity of RNase A could be associated with the increased presence of pre-folded intermediate forms. As we expected, the activity assay showed 3.3-fold reduction of RNase A produced in the periplasm of Δ*dsbC* compared to the one produced in the *wt* strain (Additional file 1: Figure S19). Going forward, protein folding modulation strategies could be coordinated (e.g., by titrating the expression or activity of folding modulators) to increase glycoprotein yield, glycosylation and to retain biological activity.

Surprisingly, the Δ*dsbC* strain expressing RNase A grew better than the *wt* strain expressing the same protein which also resulted in increased recombinant protein titre (Table 4). Both *wild-type* and Δ*dsbC* expressing NGRP showed little or no difference in the cell growth and protein titre (Table 4). We also observed that the expression of scFv13R4 but not the CM variant in the Δ*dsbC* strain negatively impacted cell growth, and consequently the recombinant protein titre (Additional file 2: Table S6). Together with the result from glycoprotein production in the Δ*dsbB* strain, we suggest different proteins could be glycosylated more efficiently in vivo based upon modulation of their distinct folding requirements to place them in a specific unfolded or intermediate form for optimal glycosylation.

**Table 4:** Comparative cell growth and volumetric production (protein titre) during RNase A production in the *wild-type* (*wt*) and Δ*dsbC* strain

Proteins	Cell growth^a^ and volumetric production (protein titre)	
	Strains	
	*wild-type* (*wt*)	Δ*dsbC*
RNase A	2.69 ± 0.05	3.60 ± 0.00
	2.94 ± 0.37 mg/L	5.43 ± 0.69 mg/L
NGRP	5.33 ± 0.09	4.96 ± 0.00
	7.79 ± 0.42 mg/L	7.95 ± 1.53 mg/L

^a^Final cell density was measured as Abs_600_ recorded 4 h after induction

^b^Total protein titre was determined from the Western blot quantification data (glycosylated and non-glycosylated), normalised and converted from sample OD_600_ of periplasmic fraction and sample volume for Western blot. For the final cell density and protein titre, the average of three biological replicates is shown and the errors indicate standard deviation. Cell culture was grown under standard cultivation condition in shake flask 10:50 ml. The change (increasing or decreasing) of cell growth and protein titre relative to control (production in *wt* strain) was analysed by t-test with Welch’s correction (Additional file 2: Table S6)

## Discussion

Our initial study with NGRP glycosylation indicated that for this particular protein, production levels had little impact upon glycosylation efficiency. Availability of lipid base, sugar precursor, and glycan conjugating enzyme could in principle be limiting factors for glycosylation, for example in the scenario where target protein production exceeds cell glycosylation capacity [33, 48, 64, 66, 69]. However, if the enzyme and lipid-glycan substrate are not rate limiting and protein glycosylation efficiency is observed similarly across different expression levels, we propose that protein glycosylation in this context could depend predominantly upon sequon accessibility to the glycosylation enzyme (PglB/oligosaccharyltransferase). In this simple way, if the sequon is not accessible for glycosylation once target protein has folded or diffused away from the membrane, the degree of glycosylation will only be dependent upon the transient unfolded state or membrane residency time during translocation, in which PglB “sees” the sequon and not the level of target protein production.

To explore the effect of membrane residency time upon protein glycosylation, we monitored the glycosylation state of proteins located in the periplasm and inner membrane. Using NGRP and scFv13R4 as the model proteins, in which the target protein was trapped in the membrane by generating a poorly processed signal cleavage site (TMT variant) attached to the target proteins. Improvement in glycosylation efficiency of TMT-scFv13R4, located in the membrane fraction, over the wt-scFv13R4 located in the periplasm fraction, seemed to agree with the hypothesis in this study. By increasing membrane residency of target protein scFv13R4, glycosylation efficiency was improved by up to 25–40%. Following extended time in the membrane, local interaction of the protein substrate and the OTase would likely increase, leading to enhanced glycosylation efficiency. However, the effectiveness of this strategy seems to be protein specific, as some factors such as protein size and structural complexity might also influence protein folding rate during membrane retention, and therefore influence sequon accessibility for glycosylation. For example, NGRP is a small protein with a predicted structure consisting of only a single α-helical chain (*d* =  ~ 10–12 Å). For this protein, folding could be initiated very early whilst inside the translocon tunnel (*d* =  ~ 20 Å) (Fig. 2A, i) [100, 101]. Previous studies showed that some proteins with more complex architecture and complex tertiary structural modifications, such as disulphide-bonds, underwent folding suppression within the translocon [84, 85]. Moreover, for many of these proteins, folding would continue for some period after translocation [102–104]. Taken together, we assume that simpler proteins like NGRP do not experience the same spatial and structural constraints as scFv13R4 upon folding in the translocon during extended residency time. These constraints suggest that, scFv13R4 is likely to remain unfolded during membrane retention, meaning that the sequon remains available for glycan addition for a greater length of time, resulting in greater interaction with OTase and more efficient glycosylation.

We then further investigated if modulation of target protein folding could enhance protein glycosylation. By monitoring the glycosylation efficiency of model disulphide bond-containing proteins, we found that producing the protein under suboptimal condition for disulphide-bond formation could enhance glycosylation efficiency. In this experiment, the rate of disulphide-bond synthesis was adjusted by varying the oxidants (oxygen, chemical oxidants, oxidoreductases DsbAB and DsbC) through changing culture conditions and *E. coli* production strain genetic backgrounds. Owing to the structural diversity of model disulphide bond-containing proteins, we also identified specific environmental and biochemical conditions to optimise glycosylation efficiency of different proteins. Sequon location was shown to play a key role is deciding which methodology is most likely to result in improved glycosylation efficiency. If the sequon is located around highly structured regions this can lead to reduced glycosylation efficiency, as shown previously [23, 71, 105, 106]. For example, we show that when located near residues involved in disulphide-bond formation, the sequon is less well glycosylated by PglB. For scFv13R4CM where the sequon is proximal to these regions, expression in the Δ*dsbB* strain may provide an extension in the time required for disulphide-formation, therefore increasing the sequon exposure for glycan transfer by PglB, leading to ~ 1.6-fold (from ~ 50% to 80%) enhanced glycosylation efficiency (Figs. 6B and 7B). In another example, RNase A glycosylation efficiency was improved ~ 1.9-fold (from ~ 28% to 53%) when the protein was produced in a Δ*dsbC* strain (Fig. 7E). As a protein with multiple disulphides, RNase A production in the Δ*dsbC* strain may fold into a specific intermediate form, leading to the sequon becoming more accessible for PglB compared to the one produced in Δ*dsbB* or under oxygen-depleted conditions. Lastly, scFv13R4 glycosylation efficiency was observed to be less sensitive, and displayed relatively similar levels across all the different disulphide attenuation treatments (1.2- to 1.3-fold or from ~ 65% to 80%). Having a distal location from cysteine residues, the availability of the R4 sequon may be less influenced by disulphide-bond formation and hence a limited effect upon interaction with PglB.

Production of disulphide bond-containing glycoproteins under these suboptimal conditions required for their folding could be problematic in terms of achieving high glycoprotein yields and titres. Indeed, cell growth was dramatically reduced during cultivation in oxygen-depleted conditions, possibly due to general metabolic burden caused by oxygen limitation. On the other hand, fitness issues during protein production in the Δ*dsbB* strain seemed to be related to protein specific stress conditions, since strains producing the control protein NGRP grew similarly under these conditions. While increased glycosylation efficiency was observed during the production of disulphide-bond containing protein RNase A in the Δ*dsbC* strain, the produced proteins were shown to be less active. Total production of disulphide bond-containing proteins was reduced 50–90% when expressed in the Δ*dsbB* strain. However, supplementation with the chemical oxidant cystine, was shown to rescue protein yield and cell viability whilst maintaining glycosylation efficiency of the Δ*dsbB* strain, therefore increasing protein titre and the absolute amount of glycoprotein produced. This result indicated the potential for using different oxidants or to tune expression levels of the native oxidoreductase to modulate folding-dependent glycosylation of target proteins and to balance with host cell physiology. To further optimise the strategy, improvement in total protein production, glycosylation efficiency, protein activity, and the effect upon the cell growth need to be considered. Therefore, those studies will be informative for the selection of strains, medium, cultivation conditions, and bioprocess design for glycoprotein production. This includes options to separate biomass production and target protein expression to avoid the physiological burden of unfolded protein stress during cell growth and to improve volumetric production (protein titre). However, it is also worthy of mention that for some production strategies, that enhanced glycosylation efficiency can be a main consideration, especially if protein purity is essential and the downstream protein purification step can be a bottleneck [48]. Moreover, if the amount of protein needed for an application is rather low, for example for use as a glycoconjugate vaccine (25–100 μg needed per vaccination), improved glycosylation efficiency may compensate for the reduction in total protein production titres [48].

**Fig. 8:**
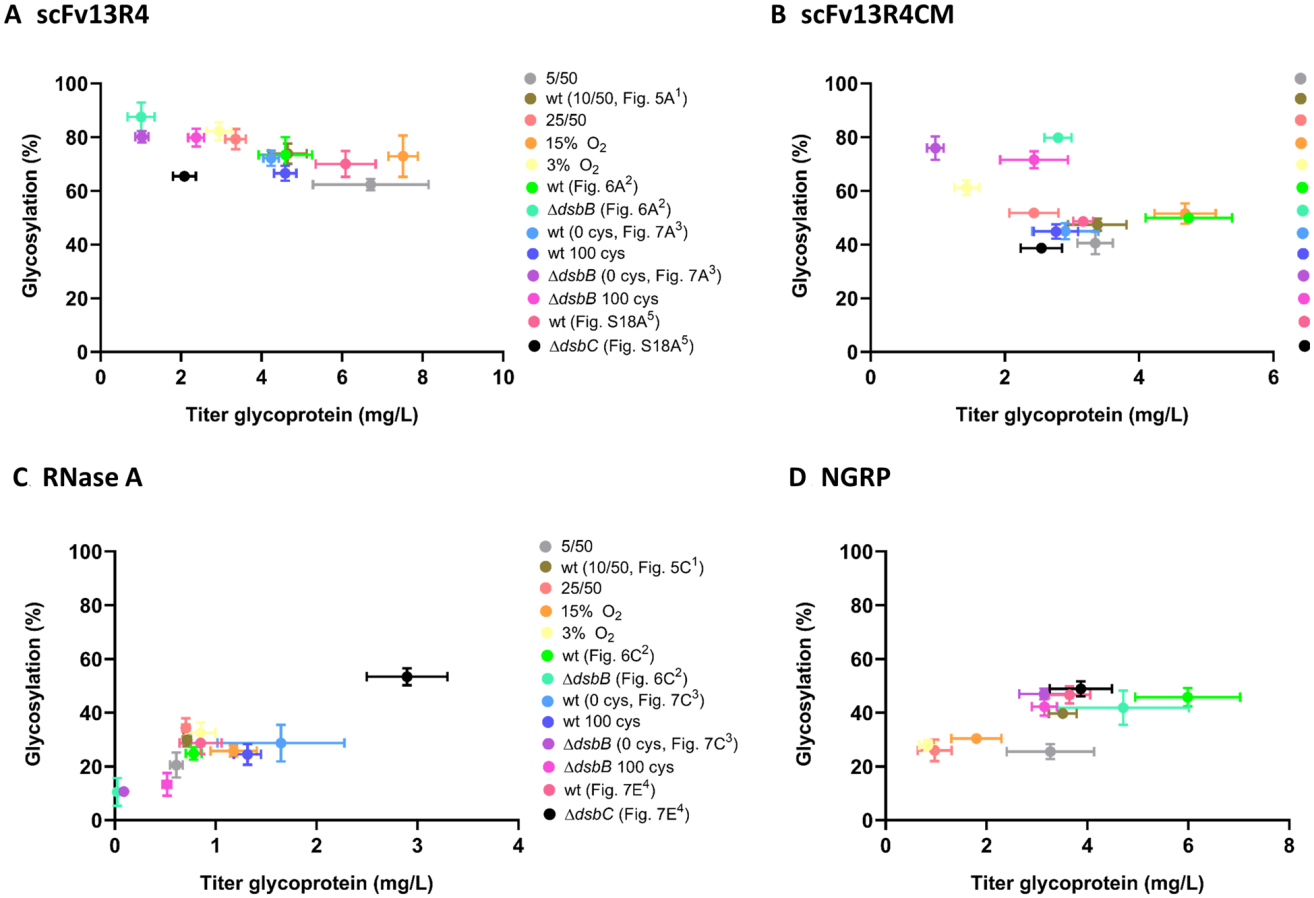
Summary of glycosylation efficiency (%) and glycoprotein titre (mg/L of glycosylated protein) of **A** scFv13R4, **B** scFv13R4CM, **C** RNase A, and **D** NGRP during production in glyco-competent *E. coli* K-12 by use of different cultivation conditions to modulate target protein folding. Glycoprotein titres were converted from the yield (mg/g DCW), cell growth, and total protein titre results given in the Figs. 5, 6, 7, and Tables 1, 2, 3, 4 and Additional file 2: Table S6 (“Methods” section). Colour symbols indicate the experiment or figure/table sources for the data (Fig; ^1^ = Table 1, ^2^ = Table 2, ^3^ = Table 3, ^4^ = Table 4, ^5^ = Additional file 2: Table S6). A similar *wt* and Δ*dsbB* cultivation were run in four different batches of experiment, which were (i) *wt* oxygen transfer experiment (10/50, Fig. 5A–D), (ii) *wt* against oxidoreductase knockout Δ*dsbB* (Fig. 6A–D), (iii) wt and ΔdsbB 0 cystine treatment (Fig. 7A–D), (iv) *wt* against oxidoreductase knockout Δ*dsbC* (Fig. 7E, F, Additional file 1: Figure S18A, B). A variation of inducer concentration for NGRP expression was used in the experiment of Fig. 6D (200 μM instead of 40 μM PPDA, “Methods” section)

As the use of different folding attenuation conditions to produce the model proteins in this study showed different pros and cons in terms of protein glycosylation efficiency, yields, and cell growth. The production strategy could also be prioritised dependent on the ultimate application and the requirement for maximising the total amount of glycoprotein (titre) vs. maximising the proportion of glycosylated product (glycosylation efficiency). The highest glycoprotein titre of the scFv model proteins used here tended to be achieved by production in the wild-type strain and under more oxygenated conditions (Fig. 8A, B). On the other hand, for production of scFv13R4 and scFv13R4CM, enhanced glycosylation efficiency was obtained by production in the Δ*dsbB* strain, in which improved titre could be achieved by cystine supplementation during the cultivation (Fig. 8A, B). Production of RNase A in the Δ*dsbC* strain resulted in increased glycosylation efficiency and glycoprotein titre, while at cost of reduced protein activity (Fig. 8C, and Additional file 1: Figure S19). Future study could explore how optimisation of folding modulators could benefit glycosylation efficiency, glycoprotein titre, and maintain biological activity of the product. Production of glycosylated NGRP in terms of glycosylation efficiency seemed to give similar results irrespective of production in the wild-type or oxidoreductase strains (Fig. 8D). The optimised condition for production of glycosylated NGRP was flask cultivation at a 10/50 culture to flask volume ratio, which gave improvement both in glycoprotein titre and glycosylation efficiency, regardless the type of producer strains (wt/Δ*dsbB*/Δ*dsbC*) (Fig. 8D). It is worthy of mention that increased NGRP glycoprotein yield and titre was achieved at higher induction expression (200 μM PPDA at the experiment in the Fig. 6D compared to 40 μM PPDA in any other experiments, “Methods” section) without significantly affecting glycosylation efficiency (Fig. 8D).

The final steps of protein translocation through the secretion machinery and subsequent folding could be viewed as a competing reaction with glycosylation, since the sequon will potentially be less available for glycan transfer upon target protein folding. In eukaryotic cells and the bacterium *C. jejuni*, glycosylation is coordinated temporally and spatially with protein translocation and folding pathways to increase glycosylation efficiency, and further has been mechanistically demonstrated in higher eukaryotic (mammalian) cells [22, 26]. This is demonstrated by coupling of transmembrane OTase activities with Sec translocation. The subunits of some eukaryotic heteroligomeric Otases possess oxidoreductase activity which interfere with disulphide-bond formation of the target proteins, leading to formation of transient intermediate protein conformations more accessible for glycosylation [71, 95]. However, expression of heterologous glycosylation machinery in *E. coli*, such as the *pgl* pathway from *C. jejuni*, appears to be uncoordinated and less efficient at protein glycosylation than in the native host. One explanation for this reduced glycosylation efficiency of the heterologous host is the potential lack of accessory proteins for modulating the translocation process and protein folding environment thereby affecting target protein sequon accessibility for the OTase [26]. Informed by glycosylation events in the native system, efforts to modulate sequon interaction with PglB could be a potential approach to optimise glycoprotein production in *E. coli*, the global aim of this present study.

Based on the general principle of folding-dependent glycosylation, the strategy performed here could be similarly adopted by using specific conditions for the production of non-disulphide bond-containing proteins. Recently, DeLisa and co-workers [92] demonstrated the versatility of cell-free glycoprotein production systems for rapid screening of functional Otases and glycosyltransferases from different bacteria and eukaryotes. Due to the open nature and modularity of this platform, screening could be rapidly and simply expanded using collections of chaperones and folding catalysts to improve glycosylation of new target proteins. Incorporating native or engineered chaperone candidates from *E. coli* or even *C. jejuni* may lead to identification of accessory proteins promoting more efficient protein glycosylation [107–110]. Moreover, the rapid characterisation of folding modulator-target protein combinations in vitro would allow early validation before further optimisation in vivo.

Lastly, the study presented here also may also show the potential of increasing membrane residency time of the target protein during Sec translocation to enhance glycosylation efficiency. While this study provides a proof of concept for this strategy, the use of uncleaved signal peptide would provide practical challenges in the downstream process of protein production, such as membrane purification and signal peptide removal. For further work, the use of alternative protein delivery mechanisms to the periplasm via the signal recognition particle (SRP) pathway may provide further opportunities to coordinate translation, translocation and glycosylation [24]. Since translocation occurs co-translationally via the SRP, modifying codon usage of the gene encoding the target protein could be used to influence transport rate [111], and hence glycosylation efficiency.

## Conclusion

In this study, we demonstrated that improved glycosylation efficiency in the heterologous host could be achieved by mimicking the coordination between protein translocation, folding and glycosylation observed in native such as *Campylobacter jejuni* and mammalian hosts. Furthermore, it provides insight into strain engineering and bioprocess strategies, to improve glycoprotein yield, titre and to avoid physiological burden of unfolded protein stress to cell growth. In addition to existing strategies to enhance protein glycosylation in *E. coli*, the work presented here provides new approaches that can be used in order to develop more robust recombinant glycoengineering platforms.

## Methods

### Strains, plasmids, and general growth conditions

Cloning strains: *E. coli* NEB5α (NEB). Expression strain: *E. coli* Top10F’ (Thermo Fisher); *E. coli* K-12 *wild -type* (*wt*), *dsbB::kan* (Δ*dsbB*) and *dsbC::kan* (Δ*dsbC*) with inactivated *dsbB* or *dsbC* due to insertion of a kanamycin-resistance gene were used as indicated (Keio collection) [112]. Plasmids: Four genes encoding target proteins; Cj0114 NGRP, anti-β-galactosidase single-chain Fv scFv13R4 and R4CM, and bovine pancreatic ribonuclease RNase A, all modified with N-terminal PelB signal peptide, C-terminal hexahistidine (His_6_) tag, and single bacterial glycosylation sequon were synthesised by gBlocks (Integrated DNA Technologies). The genes were subcloned into ampicillin resistance determinant-containing pDEST-ORS expression vector [76]. Chloramphenicol resistance determinant-containing pACYC184 plasmid carrying the entire *C. jejuni* pgl locus (pACYC*pgl*) [15] was transformed into the expression strain to allow glycoprotein production (glyco-competent). General growth conditions: *E. coli* NEB5α strains were routinely grown in LB medium (0.5% yeast extract, 0.5% NaCl, 1.0% Bactotryptone) to enrich constructed plasmids. For glycoprotein production studies, expression strains of *E. coli* were grown in LB medium as starter cultures and in TB medium (2.7% yeast extract, 4.5% glycerol, 1.3% Bactotryptone) with 0.2% glucose for protein expression. All media were supplemented with antibiotics as required (ampicillin 100 μg/ml, kanamycin 50 μg/ml, and chloramphenicol 25 μg/ml). Protein expression was induced by the addition of IPTG (Isopropyl β-d-1-thiogalactopyranoside) and PPDA (Pyrimido [4,5-d] pyrimidine-2,4-diamine) as indicated.

### In silico analysis of protein structure and translocation

Homology modelling of NGRP, scFv13R4, and scFv13R4CM was performed using Phyre2 (protein homology/analogy recognition engine) [113]. Ribbon models were drawn by UCSF Chimera [114]. Signal peptide profile and protein translocation of NGRP/scFv13R4 wt and TMT were predicted using SignalP4.1 [81].

### PCR site-directed mutagenesis

Primers used to generate mutant versions of the PelB cleavage site (TMT) are listed in Additional file 2: Table S1 and were supplied by Integrated DNA Technologies. Inserts NGRP or scFv13R4 and linearised plasmid (backbone) pDEST-ORS-PelB TMT mutant were generated from the template pDEST-ORS-PelB wt NGRP or scFv13R4 by PCR: 98 °C for 30 s; 35 cycles of 10 s at 98 °C, 30 s at the primer annealing temperature (gradient), and extension at 72^o^Cfor 30 s per 1 kb; with a final extension step of 72 °C for 10 min. PCR products were treated with DpnI (NEB) and purified using a gel extraction kit (Qiagen). Insert NGRP or scFv13R4 was assembled into backbone pDEST-ORS-PelB TMT following NEB builder Hi-fi mix protocol (NEB) to create pDEST-ORS-NGRP/scFv13R4 TMT. Plasmid products were cloned into *E. coli* NEB5α and isolated by miniprep according to the manufacturers (Qiagen) protocol. All constructs were confirmed by DNA sequencing.

### Growth conditions for glycoprotein production

To prepare inocula for glycoprotein production tests, expression strains of *E. coli* bearing pACYC*pgl* (glyco-competent) were transformed with pDEST-ORS constructs specific for each target protein. Freshly plated colony transformants were picked and grown overnight (37 °C, 200 rpm) in LB medium supplemented with antibiotics. The starter culture then was transferred (1% v/v) into TB medium (with added glucose and antibiotics) in a shake flask and grown to OD_600_ = 0.6 (37 °C, 200 rpm). At this point, cells were induced with IPTG (100 μM) and PPDA (0–400 μM or 0–200 μM for titration assay; 400 μM during membrane residency test of wt and TMT variant; 40 μM for NGRP during expression in oxygen-depleted conditions, in Δ*dsbC* strain, and in Δ*dsbB* strain with cystine supplementation; 200 μM for NGRP during expression in Δ*dsbB* strain; 0 μM for scFv13R4 and scFv13R4CM, and 200 μM for RNase A in all conditions for redox attenuation experiment) and then divided into three for different cultivation strategies. Microculture: 1 ml of cultures in 96-well deep well plate, 37 °C 1000 rpm for 3 h (titration assay) or 6 h (membrane residency test of wt and TMT variant). Shake flask: 10 ml (standard condition) or 5, 10 and 25 ml (treatment conditions, as indicated) of culture in 50 ml shake flask, 37 °C 200 rpm for 4 h or 6 h (titration assay of wt and TMT variant only). 6-well: 5 ml of cultures in 6-well plate, incubated in a Don Whitley VA500 microaerobic cabinet set to 3% or 15% O_2_, 37 °C 100 rpm for 4 h.

### Cell fractionation

Cell fractionation was performed from the final culture of glycoprotein production experiments after the specified time post-induction. Cultures were normalised to the same ODV 1–5 (OD_600_ x ml = 1–5) to collects periplasmic and membrane fractions. The cultures with specific ODV were centrifuged (17,000*g*, 4 °C, 1 min) and cell pellets resuspended in 250 µl Buffer 1 (500 mM Sucrose, 5 mM EDTA, 100 mM Tris–acetate pH 8.2), followed by addition of lysozyme (0.16 mg/ml) and Milli-Q water (250 μl). Cells were incubated on ice for 5 min, and 10 µl of 1 M MgSO_4_ added to stabilise the spheroplast. Periplasm (supernatant) was collected from spheroplast (pellet) by centrifugation (17,000*g*, 4 °C, 10 min). To produce membrane fractions, spheroplasts were washed with 500 µl Buffer 2 (250 mM sucrose, 10 mM MgSO_4_, 50 mM Tris–acetate pH 8.2), resuspended in 500 µl Buffer 3 (2.5 mM EDTA, 0.1% sodium deoxycholate, 2U benzonase, 50 mM Tris–acetate pH 8.2), and stored overnight at − 80 °C. The following day, spheroplasts were lysed by freeze-thawing and cytoplasm (supernatant) and membrane fractions (pellet) separated by centrifugation (17,000*g*, 4 °C, 30 min). Pellets were washed with 500 μl Buffer 3 and resuspended in 500 μl of Buffer 3 + SDS (1%) to obtain the membrane fraction.

### Western blot analysis

The amount of glycoprotein produced in periplasmic and membrane fractions was analysed by Western blot. Samples were resuspended in SDS-PAGE loading buffer (ThermoFisher), supplemented with 75 mM dithiothreitol, and boiled for 10 min. Equal amounts of sample were separated by SDS PAGE (Biorad) and Western blotted onto PVDF 0.2 µM pore size membrane using a Turbo Transblot apparatus (Biorad). The membrane was blocked with 5% (w/v) milk powder in PBS (30 min, 50 rpm shaking, room temperature). Membranes were incubated overnight (50 rpm, 4 °C) with mouse monoclonal anti-His antibody (1:3000 dilution in PBS 5% milk, Pierce) and rabbit polyclonal anti-Cj glycan (CjNgp) primary antibody(1:500 dilution in PBS 5% milk) as appropriate. Membranes were washed three times with PBS, followed by incubation with anti-mouse and anti-rabbit secondary antibody (1:30,000 dilution in PBS 5%, Li-Cor) (50 rpm, in the dark at room temperature, 30 min), and then washed three times with PBS. The protein bands were visualised with a Li-Cor Odyssey scanner. Proteins were quantified based on densitometry analysis using Li-Cor Image Studio 5.0. Pre-determined purified protein from each sample (scFv13R4, RNase A, or NGRP) was used as a standard for quantification (5 ng to 100 ng). All data were produced in biological triplicate.

### Data processing and statistical analysis

Data were processed and analysed using Microsoft Excel and GraphPad Prism9. Each data point was the average from three biological replicates, and error bars show calculated standard deviation. For Western blot quantification, protein samples were measured from each biological replicate of isolated cell fraction (periplasmic or membrane). Additional file 1: Figure S20 shows a representative Western blots images for blot reproducibility of PelB 2-NGRP (representative from Fig. 3C) expressed in periplasmic fraction from three biological replicates. A standard curve was constructed from pre-determined purified protein (scFv13R4, RNase A, or NGRP: 5–100 ng), and used to calibrate Western blot signal intensity of protein samples. The data was fitted with linear regression, and the linear equation from the standard curve was used to normalise and convert the signal into ng of the protein. Representative standard curve for NGRP and the use of standard curve for protein quantification are shown in Additional file 1: Figure S21. Conversion of protein quantification data into the yield mg/g of dry cell weight (DCW) based on normalisation and calculation from the measured OD_600_ of samples. Dry cell weight was determined following the previous protocol [76], and the conversion factor of 0.35 mg/ml per OD_600_ was acquired. Protein titre mg/l was determined from protein quantification data (ng) converted and normalised from OD_600_ of collected samples and and volume of sample (μl) for Western blot. Statistical analysis was conducted by unpaired t-test with Welch’s correction, and P value < 0.05 was considered statistically significant.

### scFv13R4 binding assay

scFv13R4 binding activity against its cognate antigen β-galactosidase was performed following bio-dot protocol [115]. 2 μl of β-galactosidase (0.3 mg/ml) dots were spotted onto a nitrocellulose membrane (Amersham Hydrobond), and the membrane then left to dry for 5–15 min. To block non-specific binding site, the membrane was incubated with 5% (w/v) milk powder in PBS for 1 h (50 rpm, RT). The membrane then was left to dry for 2 h in 40 °C. To prepare binding mix, each 2 μl of protein sample from isolated periplasmic fraction was added onto the dot of β-galactosidase, with 2 μl of protein buffer was added instead as a negative control. The membrane then left to dry again for 15 min. Afterwards, the membrane was probed with primary (mouse monoclonal anti-his) and secondary (anti-mouse) antibody to detect the scFv13R4 following the similar protocol with the Western blot. The protein dots were visualised with the Li-Cor Odyssey scanner and the signal intensity was analysed by densitometry using Image Studio 5.0 Software. Specific binding activity of was measured by normalising the densitometric signal with the amount of protein sample. All data were produced in biological triplicate.

### RNase A activity assay

RnaseAlert lab test kit (Invitrogen) was used to assay RNase A activity of the protein sample. The reaction mix consist of 10 μl of RNase A substrate (20× dilution with RNase A buffer solution), 5 μl of protein sample from periplasmic fraction (1000×), and 85 μl of nuclease-free water. As a negative control, 5 μl of protein buffer or 5 μl of protein sample from periplasmic fraction of non RNase A producer strain was added instead into the reaction mix. The reaction mix was transferred into 96-well plate, and then incubated in the Clariostar plate reader for 1 h 37 °C. The RNase A substrate from the kit is a modified RNA nucleotide that emits a green fluorescence when it is degraded by RNase. The fluorescence activity of degraded substrate was recorded overtime during the incubation (RFU at 490/520 nm). RNase A specific activity was quantified by normalising the rate of substrate degradation (RFU/minute) with the amount of protein sample. All data were produced in biological triplicate.

## Supplementary Information

**Additional file 1.** Additional figures

**Additional file 2: Table S1.** Primers used in PCR site-directed mutagenesis to generate TMT mutant of PelB NGRP and scFv13R4 in pDEST-ORS construct. **Table S2.** The effect of oxygen transfer conditions (culture to flask volume ratio) to cell growth and protein titre (mg/L) during production of model disulphide-bond proteins in glyco-competent *E. coli* Top10F’. **Table S3.** The effect of oxygen level conditions (15% and 3% O_2_) to cell growth and protein titre (mg/L) during production of model disulphide-bond proteins in glyco-competent *E. coli* Top10F’. **Table S4.** The effect of *dsbB* knockout to cell growth and protein titre (mg/L) during production of model disulphide-bond proteins in glyco-competent *E. coli* Top10F’. **Table S5.** The effect of cystine supplementation to cell growth and protein titre (mg/L) during production of model disulphide-bond proteins in glyco-competent *E. coli* Top10F’ *wt* and Δ*dsbB* strains. **Table S6.** The effect of *dsbC* knockout to cell growth and protein titre (mg/L) during production of model disulphide-bond proteins in glyco-competent *E. coli* Top10F’.

## Data Availability

All data generated or analysed in this study are included in this manuscript or in Additional files. Raw data are available from the corresponding author upon reasonable request.
