# Correction to: Genetic and process engineering strategies for enhanced recombinant *N*-glycoprotein production in bacteria

**DOI:** 10.1186/s12934-021-01700-5

**Published:** 2021-11-12

**Authors:** Fenryco Pratama, Dennis Linton, Neil Dixon

**Affiliations:** 1 Manchester Institute of Biotechnology (MIB), The University of Manchester, Manchester, M1 7DN UK; 2 Department of Chemistry, The University of Manchester, Manchester, M1 7DN UK; 3 Faculty of Biology, Medicine and Health, The University of Manchester, Manchester, M1 7DN UK; 4 Microbial Biotechnology Research Group, School of Life Sciences and Technology, Institut Teknologi Bandung, Bandung, 40132 Indonesia

## Correction to: Microb Cell Fact (2021) 20:198 10.1186/s12934-021-01689-x

Following publication of the original article [1], the authors identified an error in Fig. 8. The correct figure (Fig. 8) is given in this correction.

**Fig. 8:**
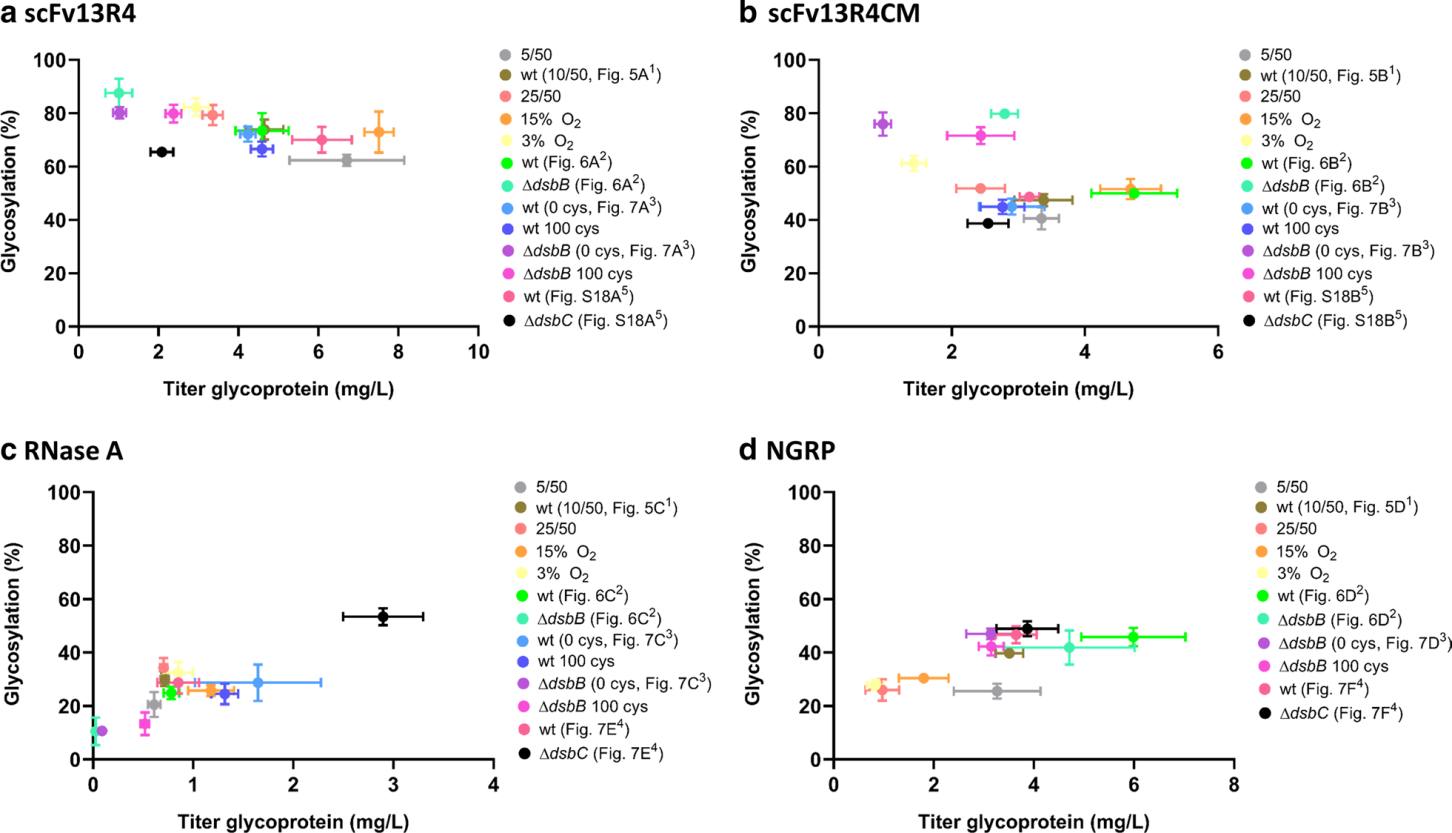
Summary of glycosylation efficiency (%) and glycoprotein titre (mg/L of glycosylated protein) of **A** scFv13R4, **B** scFv13R4CM, **C** RNase A, and **D** NGRP during production in glyco-competent* E. coli* K-12 by use of different cultivation conditions to modulate target protein folding. Glycoprotein titres were converted from the yield (mg/g DCW), cell growth, and total protein titre results given in the Figs. 5, 6, 7, and Tables 1, 2, 3, 4 and Additional file 2: Table S6 (“Methods” section). Colour symbols indicate the experiment or figure/table sources for the data (Fig; ^1^ = Table 1, ^2^ = Table 2, ^3^ = Table 3, ^4^ = Table 4, ^5^=Additional file 2: Table S6). A similar *wt* and Δ*dsbB* cultivation were run in four different batches of experiment, which were (i) *wt* oxygen transfer experiment (10/50, Fig. 5A–D), (ii) *wt* against oxidoreductase knockout Δ*dsbB* (Fig. 6A–D), (iii) wt and ΔdsbB 0 cystine treatment (Fig. 7A–D), (iv) *wt* against oxidoreductase knockout Δ*dsbC* (Fig. 7E, F, Additional file 1: Figure S18A, B). A variation of inducer concentration for NGRP expression was used in the experiment of Fig. 6D (200 μM instead of 40 μM PPDA, “Methods” section)
