# Supplementary material for: Genetic and process engineering strategies for enhanced recombinant N-glycoprotein production in bacteria
Source: Microb Cell Fact. 2021 Oct 14;20:198. doi: 10.1186/s12934-021-01689-x (PMC8518210; doi:10.1186/s12934-021-01689-x)
Supplement: Supplementary file 1 — Additional file 1. Additional figures [file 12934_2021_1689_MOESM1_ESM.docx]

**Genetic and process engineering strategies for enhanced recombinant N-glycoprotein production in bacteria**

# Fenryco Pratama^1,2,4^, Dennis Linton^3^, Neil Dixon^1,2^

# ^1^Manchester Institute of Biotechnology (MIB), ^2^Department of Chemistry, ^3^Faculty of Biology, Medicine and Health, The University of Manchester, Manchester, M1 7DN, UK. ^4^Microbial Biotechnology Research Group, School of Life Sciences and Technology, Institut Teknologi Bandung, Bandung, 40132, Indonesia.

# Correspondence: [neil.dixon@manchester.ac.uk](mailto:neil.dixon@manchester.ac.uk)

Additional file 1

## Figure S1. (A) Expression of NGRP from pDEST-ORS construct under no inducer (uninduced or UI), IPTG only (100 μM), and IPTG (100 μM) + 400 μM PPDA induction. Representative blot from PelB 1-NGRP titration assay is shown. The lane in figure A is from the same blot while a white vertical line indicates a non-adjacent lane. The protein migration band relative to each other in different lanes in the blot is unchanged. (B-D) Titrability test of PelB-NGRP variants (WT, PelB 1 and PelB 2) expressed from pDEST-ORS constructs. sfGFP was fused at C-terminal of NGRP to monitor NGRP expression based on fluorescence assay (FITC-A compensated) in flow cytometry. Induction conditions are indicated.

## Figure S2. Correlation analysis (Pearson) between glycosylation and total protein level of NGRP produced from the pDEST-ORS-PelB 1 (A) and PelB 2-NGRP (B) construct in glyco-competent *E. coli* Top10F’ over different inducer concentration (100 μM IPTG and 0, 2, 8, 40, 100, 200, 400 μM PPDA). All data were processed from three biological replicates. Error bars indicate standard deviation from mean values. *P* < 0.05 is considered statistically significant.

## Figure S3. Cell growth comparison (Final OD_600_) of glyco-competent *E. coli* producing the target proteins containing leader sequence variant (A) PelB 1 or (B) PelB 2-NGRP across different inducer levels. NGRP was produced from the pDEST-ORS construct under IPTG only (100 μM, 0 μM PPDA), and IPTG (100 μM) + PPDA induction at different concentration (2, 8, 40, 100, 200, 400 μM). (C and D) Correlation analysis (Pearson) between cell growth (final OD_600_) and total protein level of NGRP produced from the pDEST-ORS-PelB 1 (C) and PelB 2-NGRP (D) constructs in glyco-competent *E. coli* Top10F’ over different inducer concentration (*P* < 0.05^*^). All data were processed from three biological replicates. Error bars indicate standard deviation from mean values.


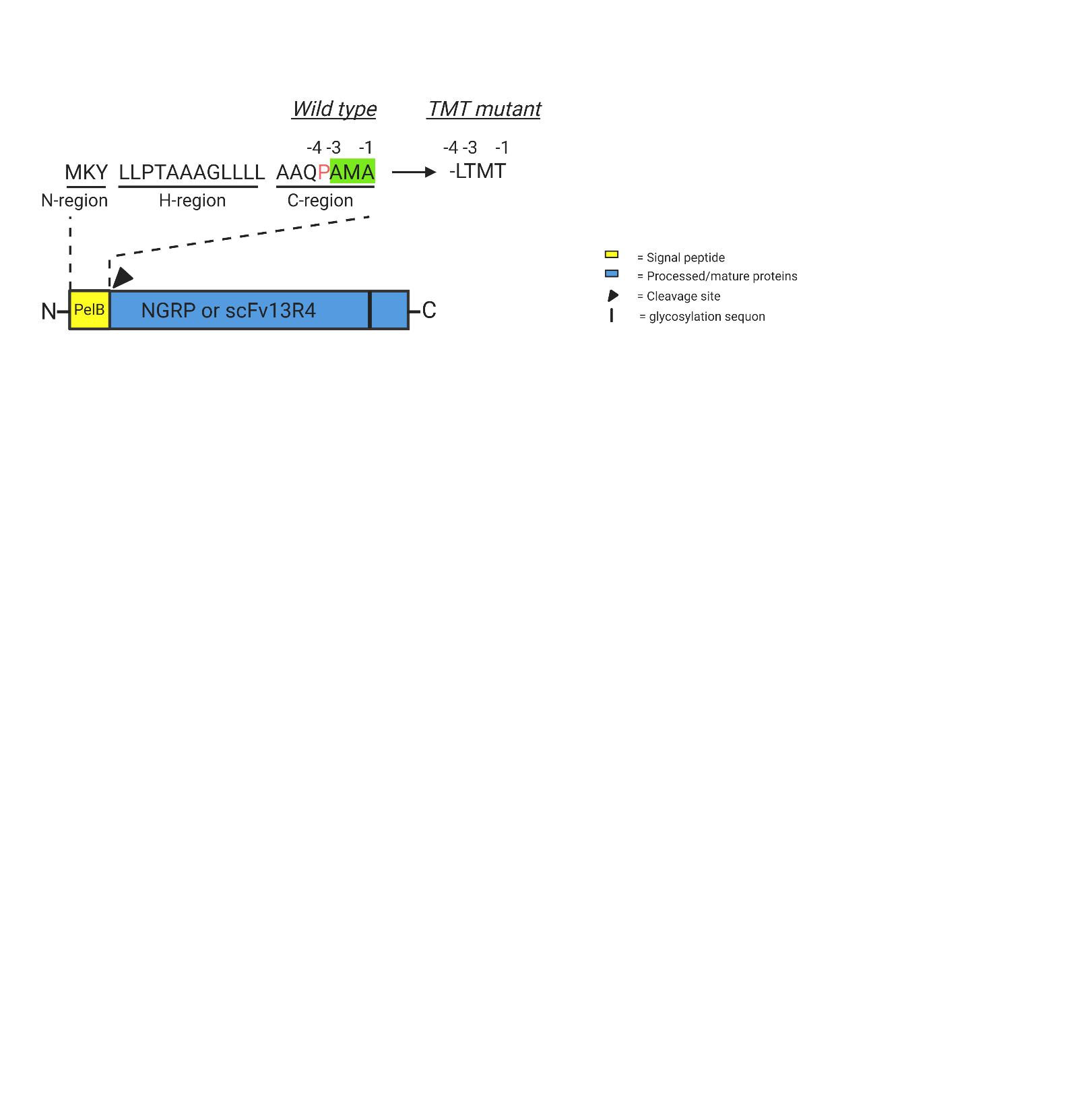


## Figure S4. Design of signal peptide cleavage site mutant of the PelB-Target protein fusion. The architecture of protein-signal peptide used in this study. Each protein (NGRP and scFv13R4) is tagged with 22-amino acids of PelB signal peptide (Sec-dependent) at the N-terminal. Three signature regions of PelB Sec peptide: N (positive residues), H (hydrophopic residues), and C-region (neutral residues) are shown. Cleavage site motif (green) and the position of its key residues (-1, -3) from the site are highlighted. Proline (red) is frequently found around the motif. Signal peptide modification from wild type (wt, P-A-M-A) and the mutant (TMT, L-T-M-T) are indicated. Each protein contains one glycosylation sequon for single glycan attachment in their structure.

| **Protein** | **Signal peptide** | | | |
| --- | --- | --- | --- | --- |
|  | **wt** | | **TMT** | |
|  | **Identity (Sec)** | **Cleavage site** | **Identity (Sec)** | **Cleavage site** |
|  | **Mean S-score** | **Y-score** | **Mean S-score** | **Y-score** |
| NGRP | 0.921 | 0.886 | 0.900 | 0.353 |
| scFv13R4 | 0.938 | 0.884 | 0.868 | 0.570 |

## Figure S5. SignalP4.1 analysis of the PelB-NGRP and -scFv13R4 carrying cleavage site mutation. (A) wt-PelB with NGRP and (B) TMT-PelB with NGRP, (C) wt-PelB with scFv (D) TMT-PelB with scFv13R4. Cleavage site motif (green bar) was modified from P-A-M-A in the wild type (wt) to L-T-M-T in the mutant. C and Y score indicate cleavage site recognition by SPaseI. S-score represents Sec signal peptide identity.


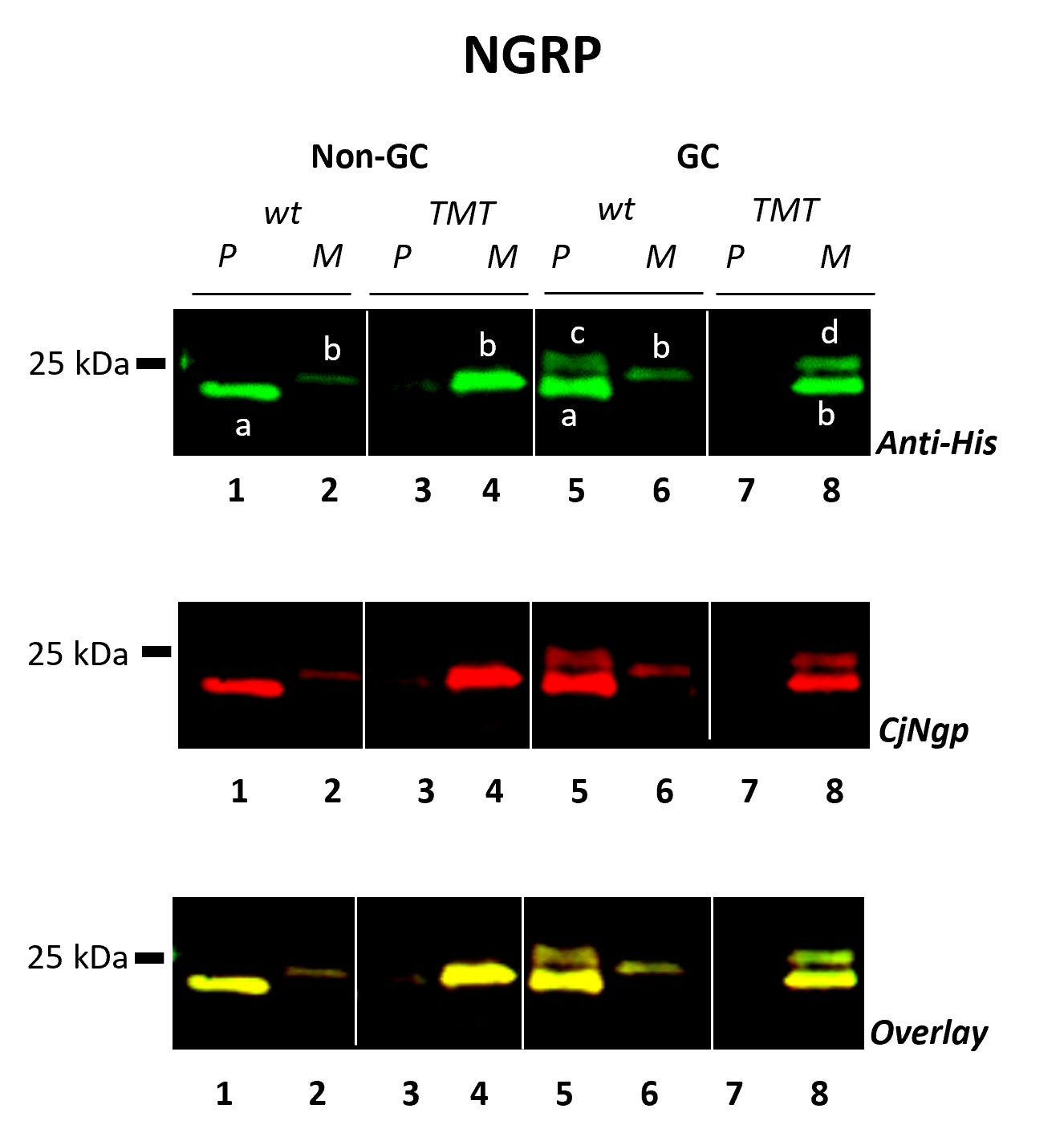


## Figure S6. Western blot analysis of membrane (M) and periplasmic (P) expression of wild type (wt) and mutant (TMT) NGRP in glyco-competent (GC) and non glyco-competent (Non-GC) *E. coli* Top10F’. All NGRP isoforms were detected equally by anti-His and CjNgp antibody. The lanes in Western blot are from the same blot while white vertical lines indicates non-adjacent lanes (uncropped blot is shown in Figure S7). The protein migration band relative to each other in different lanes in the blot is unchanged.

## Figure S7. Uncropped Western blot image of Fig. 4B. Western blot analysis of membrane (M) and periplasmic (P) expression of wild type (wt) and signal peptide cleavage mutant (TMT) NGRP in glyco-competent (GC) and non glyco-competent (Non-GC) *E. coli*. NGRP detected by anti-His antibody. Predicted a-d isoforms within the bands are indicated.

## Figure S8. Western blot analysis of mixed sample from periplasmic (P) and membrane (M) expression of wild type (wt) and mutant (TMT) NGRP (A) and scFv13R4 (B) in glyco-competent (GC) *E. coli* Top10F’. Samples were run in the SDS gel by extended electrophoresis running time. Different migration rate of NGRP and scFv13R4 isoforms (a-d) is indicated. Gel migration at separated lanes refers to the main figure (Fig. 4, *B-D*) from sample at lane 5 and 8.

## Figure S9. Comparison of total protein production (yield) between periplasmic (wt) and membrane (TMT) fractions of (A) NGRP and (B) scFv13R4 under the same induction conditions. All data (A-B) were processed from three biological replicates. Error bars indicate standard deviation. Statistical analysis was conducted by unpaired t-test with Welch’s correction (*P* < 0.05*, < 0.01**, < 0.001***).

## Figure S10. Correlation analysis (Pearson) between glycosylation and total protein level of (A) periplasmic (per) wt and (B) membrane (mem) TMT-NGRP, and (C) periplasmic wt and (D) membrane TMT-scFv13R4 PelB 2-NGRP (B) produced in glyco-competent *E. coli* K12 over different inducer concentration (100 μM IPTG and 0, 8, 40, 200 μM PPDA) (*P* < 0.05*, < 0.01**). All data were processed from three biological replicates. Error bars indicate standard deviation from mean values.

## Figure S11. Cell growth comparison (Final OD_600_) of glyco-competent *E. coli* expressing target protein containing signal peptide cleavage site variants (A) NGRP-wt or (B) TMT, (C) scFv13R4-wt or (D) TMT across different inducer expression levels. NGRP or scFv13R4-wt or TMT was expressed from pDEST-ORS construct under IPTG only (100 μM, 0 μM PPDA), and IPTG (100 μM) + PPDA induction at different concentration (8, 40, and 200 μM).

## Figure S12. Comparison of glycosylation efficiency between wt and TMT of (A) scFv13R4 and (B) NGRP under the same induction conditions. All data (A-B) were processed from three biological replicates. Error bars indicate standard deviation. Statistical analysis was conducted by unpaired t-test with Welch’s correction (*P* < 0.05*, < 0.01**).

## Figure S13. Linear regression analysis between glycosylation efficieny (%) and protein expression level (mg/g DCW) of wt-scFv13R4 produced in glyco-competent *E. coli* K12 over different inducer concentration (100 μM IPTG and 0, 8, 40, 200 μM PPDA). All data were processed from three biological replicates. Error bars indicate standard deviation from mean values. Linear regression equation.

## Figure S14. Correlation analysis (Pearson) between glycosylation and total protein level of (A) scFv13R4, (B) scFv13R4CM, (C) RNase A, and (D) NGRP produced in periplasmic of glyco-competent *E. coli* K12 under three different culture to flask volume ratio conditions (5:50, 10:50, and 25:50 ml). (*P* < 0.05*). All data were processed from three biological replicates. Error bars indicate standard deviation from mean values.

## Figure S15. Comparison of relative glycosylation efficiency (RGE) of model disulphide bond-containing proteins to the RGE of NGRP. RGE of the proteins is calculated from the ratio of glycosylation at treatment (e.g. 3% O_2_) to glycosylation at control condition (e.g. 15% O_2_) (formula shown as inset). Statistical analysis was conducted by unpaired t-test with Welch’s correction relative to NGRP (*P* < 0.001***, < 0.0001****). All data were processed from three biological replicates. Error bars indicate standard deviation from mean values.

## Figure S16. Representative Western blot images of (A) scFv13R4CM and (B) RNase A expressed in *wt* and Δ*dsbB* strains of glyco-competent *E. coli* K-12. The lanes in Western blot scFv13R4CM (A) are from the same blot while white vertical lines indicates non-adjacent lanes. The protein migration band relative to each other in different lanes in the blot is unchanged (A).

## Figure S17. Analysis of scFv13R4 binding activity to its cognate antigen β-galactosidase by dot-blot assay. Protein was produced in the periplasm of glyco-competent *E. coli* *wild-type* (*wt*) or Δ*dsbB* strain supplemented with or without 100 μM cystine during protein expression. 2 μL of protein samples was dotted against 2 uL of 0.3 mg/mL β-galactosidase. The bound proteins were detected using Anti-His antibody. All data were processed from three biological replicates.

## Figure S18. Quantitative Western blot analysis (densitometry) of (A) scFv13R4 and (B) scFv13R4CM expressed in periplasmic of glyco-competent *E. coli* Δ*dsbC*. Total proteins (A and B) were quantified using pre-determined purified scFv13R4CM standard curve (5 ng to 75 ng). The data were converted into mg/g of dry cell weight (DCW) based on normalisation and calculation with measured OD_600_ of the samples. Glycosylated (yellow bar) and non-glycosylated (green bar) protein as shown (left y-axis). % Glycosylation (% G_1_/G_0_+G_1_) is indicated (black circle, right y-axis). Statistical analysis was conducted by unpaired t-test with Welch’s correction to control sample expressed in *wt* strain (*P* < 0.01**, for % glycosylation; *P* < 0.01^◊^, for normalised total protein). All data were processed from three biological replicates. Error bars indicate standard deviation from mean values.

## Figure S19. Analysis of RNaseA activity using RNaseAlert fluorometry assay. Substrate for the assay is a fluorophore that will be activated (excited) upon degradation by RNase. Substrate (20x dil) was mixed with the periplasmic fraction of the protein samples (1000x dil), and the reaction was run at 37C with RFU monitoring every 5 mins. Buffer solution (buffer 2) and periplasmic fraction from non RNase A producer strain (scFv13R4 producer) were used as the negative controls. All data were proceeded from three biological replicates.

## Figure S20. Representative Western blot images (blots reproducibility) of PelB 2-NGRP produced in periplasmic of glyco-competent *E. coli* K-12 under different inducer concentrations (100 μM IPTG and 0, 2, 8, 40, 100, 200, 400). Protein bands from three biological replicates (bio rep, 1-3) were shown.

 **Figure S21.** Quantitative Western blot analysis using standard curve of predetermined purified protein samples for calibration. (A) Figure representative of NGRP standard curve. A main standard curve (calibration curve) was constructed from triplicate blot of predetermined purified NGRP (15, 25, 50, 75, and 100 ng). (B) Representative blot for quantification of NGRP during production in glyco-competent *E. coli* under different oxygen level conditions during cultivation (Fig. 5D). A set of protein standard within range of main standard curve (15-100 ng) was run along with protein samples. The band intensity (WB signal) of each standard was divided to the average band intensity of standard from the same data point (protein amount) in the main curve. These values were then averaged to determine transfer efficiency of the blot. The transfer efficiency was used to convert signal of protein samples (WB signal), and the converted signals were then calibrated using linear regression equation of main standard curve to determine protein concentration (ng)**.**
