# Supplementary material for: Genetic and process engineering strategies for enhanced recombinant N-glycoprotein production in bacteria
Source: Microb Cell Fact. 2021 Oct 14;20:198. doi: 10.1186/s12934-021-01689-x (PMC8518210; doi:10.1186/s12934-021-01689-x)
Supplement: Supplementary file 2 — Additional file 2: Table S1. Primers used in PCR site-directed mutagenesis to generate TMT mutant of PelB NGRP and scFv13R4 in pDEST-ORS construct. Table S2. The effect of oxygen transfer conditions (culture to flask volume ratio) to cell growth and protein titre (mg/L) during production of model disulphide-bond proteins in glyco-competent E. coli Top10F’. Table S3. The effect of oxygen level conditions (15% and 3% O2) to cell growth and protein titre (mg/L) during production of model disulphide-bond proteins in glyco-competent E. coli Top10F’. Table S4. The effect of dsbB knockout to cell growth and protein titre (mg/L) during production of model disulphide-bond proteins in glyco-competent E. coli Top10F’. Table S5. The effect of cystine supplementation to cell growth and protein titre (mg/L) during production of model disulphide-bond proteins in glyco-competent E. coli Top10F’ wt and ΔdsbB strains. Table S6. The effect of dsbC knockout to cell growth and protein titre (mg/L) during production of model disulphide-bond proteins in glyco-competent E. coli Top10F’. [file 12934_2021_1689_MOESM2_ESM.docx]

**Genetic and process engineering strategies for enhanced recombinant N-glycoprotein production in bacteria**

# Fenryco Pratama^1,2,4^, Dennis Linton^3^, Neil Dixon^1,2^

# ^1^Manchester Institute of Biotechnology (MIB), ^2^Department of Chemistry, ^3^Faculty of Biology, Medicine and Health, The University of Manchester, Manchester, M1 7DN, UK. ^4^Microbial Biotechnology Research Group, School of Life Sciences and Technology, Institut Teknologi Bandung, Bandung, 40132, Indonesia.

# Correspondence: [neil.dixon@manchester.ac.uk](mailto:neil.dixon@manchester.ac.uk)

Additional file 2

##

## Additional files 2

## Table S1. Primers used in PCR site-directed mutagenesis to generate TMT mutant of PelB NGRP and scFv13R4 in pDEST-ORS construct

| **Primer** | **Sequence** | **Characteristics or Use** |
| --- | --- | --- |
| Primer 1 (forward)  Primer 2 (reverse) | 5’- TGGTAATATCACCCATGGTCATGGTCAGCTGTGCTGC-3’  5’- TCACCACCATCATCATTAAGATCCGGCTGCTAAC-3’ | - pDEST-ORS-NGRP TMT construct   To generate linearised pDEST-ORS-PelB TMT from pDEST-ORS-PelB wt NGRP template |
| Primer 3 (forward)  Primer 4 (reverse) | 5’- ATGGGTGATATTACCAGCAATAGCAG-3’  5’- ATGATGATGGTGGTGATGGTGATGATGATG-3’ | - pDEST-ORS-NGRP TMT construct   To generate NGRP insert from pDEST-ORS-NGRP wt template |
| Primer 5 (forward)  Primer 6 (reverse) | 5’- CACCACCATCATTAAGATCCGGCTGCTAACAAAG-3’  5’- TTCTGCCATCATATGGGTCATGGTCAGCTGTGC-3’ | - pDEST-ORS-scFv13R4 TMT construct   To generate linearised pDES-ORS-PelB9 TMT from pDEST-ORS-NGRP wt template |
| Primer 7 (forward)  Primer 8 (reverse) | 5’- CATATGATGGCAGAAGTTCAGCTG-3’  5’- GCCGGATCTTAATGATGGTGG-3’ | - pDEST-ORS-scFv13R4 TMT construct   To generate scFv13R4 insert from pDEST-ORS-scFv13R4 wt template |

## Table S2. The effect of oxygen transfer conditions (culture to flask volume ratio) to cell growth and protein titre (mg/L) during production of model disulphide-bond proteins in glyco-competent *E. coli* Top10F’. Statistical analysis was conducted using unpaired t-test with Welch’s correction (*P*-value < 0.05*, < 0.01**, < 0.001***, < 0.0001****)

| **Protein models** | **Final OD_600_** | | | ***P*-value** | | | **Protein titre (mg/L)** | | | ***P*-value** | | |
| --- | --- | --- | --- | --- | --- | --- | --- | --- | --- | --- | --- | --- |
|  | **5/50** | **10/50** | **25/50** | **5/50 vs 10/50** | **5/50 vs 25/50** | **10/50 vs 25/50** | **5/50** | **10/50** | **25/50** | **5/50 vs 10/50** | **5/50 vs 10/50** | **10/50 vs 25/50** |
| scFv13R4 | 5.49 ± 0.05 | 4.35 ± 0.17 | 2.85 ± 0.05 | 0.004** | 3.2 x 10^-7^ **** | 0.002** | 10.78 ± 2.37 | 6.30 ± 0.63 | 4.25 ± 0.49 | 0.07 | 0.04* | 0.01* |
| scFv13R4CM | 5.01 ± 0.12 | 4.13 ± 0.09 | 2.83 ± 0.13 | 8.1 x 10^-4^ **** | 2.8 x 10^-5^ **** | 2.3 x 10^-4^ *** | 8.29 ± 0.76 | 7.13 ± 1.01 | 4.72 ± 0.88 | 0.193 | 0.006** | 0.036* |
| RNase A | 3.65 ± 0.09 | 2.56 ± 0.00 | 1.84 ± 0.08 | 0.002** | 1.6 x 10^-5^ **** | 0.004** | 3.24 ± 0.49 | 3.14 ± 0.50 | 2.27 ± 0.29 | 0.8 | 0.054 | 0.076 |
| NGRP | 8.87 ± 0.46 | 5.33 ± 0.17 | 3.31 ± 0.09 | 0.002** | 0.002** | 2.7 x 10^-4^ | 12.65 ± 2.11 | 8.83 ± 0.40 | 3.66 ± 0.78 | 0.083 | 0.01* | 0.002** |

## Table S3. The effect of oxygen level conditions (15% and 3% O_2_) to cell growth and protein titre (mg/L) during production of model disulphide-bond proteins in glyco-competent *E. coli* Top10F’. Statistical analysis was conducted using unpaired t-test with Welch’s correction (*P*-value < 0.05*, < 0.01**, < 0.0001****)

| **Protein models** | **Final OD_600_** | | ***P*-value** | **Protein titre (mg/L)** | | ***P*-value** |
| --- | --- | --- | --- | --- | --- | --- |
|  | **15% O_2_** | **3% O_2_** | **15 vs 3% O_2_** | **15% O_2_** | **3% O_2_** | **15 vs 3% O_2_** |
| scFv13R4 | 5.57 ± 0.09 | 2.43 ± 0.05 | 1.8 x 10^-5^ **** | 10.40 ± 1.39 | 3.59. ± 0.45 | 0.008** |
| scFv13R4CM | 4.43 ± 0.05 | 2.34 ± 0.05 | 6.5 x 10^-7^ **** | 9.15 ± 1.39 | 2.35 ± 0.40 | 0.009** |
| RNase A | 2.93 ± 0.05 | 1.92 ± 0.00 | 6.9 x 10^-4^ **** | 4.55 ± 0.60 | 2.63 ± 0.41 | 0.014* |
| NGRP | 4.83 ± 0.17 | 2.51 ± 0.05 | 8.7 x 10^-4^ **** | 5.88 ± 1.40 | 2.99 ± 0.52 | 0.056 |

## Table S4. The effect of *dsbB* knockout to cell growth and protein titre (mg/L) during production of model disulphide-bond proteins in glyco-competent *E. coli* Top10F’. Statistical analysis was conducted using unpaired t-test with Welch’s correction (*P*-value < 0.01**, < 0.001***)

| **Protein models** | **Final OD_600_** | | ***P*-value** | **Protein titre (mg/L)** | | ***P*-value** |
| --- | --- | --- | --- | --- | --- | --- |
|  | ***wt*** | **Δ*dsbB*** | **wt vs Δ*dsbB*** | ***wt*** | **Δ*dsbB*** | **wt vs Δ*dsbB*** |
| scFv13R4 | 5.04 ± 0.14 | 3.27 ± 0.08 | 2.4 x 10^-4^ *** | 6.25 ± 0.73 | 1.16 ± 0.42 | 0.001** |
| scFv13R4CM | 5.40 ± 0.20 | 3.68 ± 0.00 | 0.004** | 9.48 ± 1.00 | 3.50 ± 0.23 | 0.007** |
| RNase A | 2.96 ± 0.08 | 3.05 ± 0.05 | 0.187 | 3.15 ± 0.14 | 0.26 ± 0.06 | 1.0 x 10^-4^ *** |
| NGRP | 4.43 ± 0.06 | 4.43 ± 0.11 | >0.999 | 13.11 ± 2.25 | 11.12 ± 1.93 | 0.312 |

## Table S5. The effect of cystine supplementation to cell growth and protein titre (mg/L) during production of model disulphide-bond proteins in glyco-competent *E. coli* Top10F’ *wt* and Δ*dsbB* strains. Statistical analysis was conducted using unpaired t-test with Welch’s correction (*P*-value < 0.05*, < 0.01**). Nd = not determined

| **Protein models** | **Final OD_600_** | | | | ***P*-value** | | **Protein titre (mg/L)** | | | | ***P*-value** | |
| --- | --- | --- | --- | --- | --- | --- | --- | --- | --- | --- | --- | --- |
|  | ***wt* 0 cys** | ***wt* 100 cys** | **Δ*dsbB* 0 cys** | **Δ*dsbB* 100 cys** | ***wt* 0 cys vs 100 cys** | **Δ*dsbB* 0 cys vs 100 cys** | ***wt* 0 cys** | ***wt* 100 cys** | **Δ*dsbB* 0 cys** | **Δ*dsbB* 100 cys** | ***wt* 0 cys vs 100 cys** | **Δ*dsbB* 0 cys vs 100 cys** |
| scFv13R4 | 4.88 ± 0.08 | 4.91 ± 0.20 | 3.08 ± 0.10 | 3.50 ± 0.05 | 0.847 | 0.009* | 5.87 ± 0.21 | 6.89 ± 0.37 | 1.28 ± 0.24 | 2.97 ± 0.14 | 0.022* | 0.001** |
| scFv13R4CM | 4.45 ± 0.09 | 4.35 ± 0.09 | 2.90 ± 0.00 | 3.65 ± 0.00 | 0.230 |  | 6.42 ± 0.70 | 6.15 ± 0.41 | 1.28 ± 0.24 | 3.42 ± 0.81 | 0.604 | 0.036* |
| RNase A | 3.55 ± 0.05 | 3.60 ± 0.14 | 3.12 ± 0.00 | 3.39 ± 0.05 | 0.581 | 0.01** | 5.64 ± 1.05 | 5.40 ± 0.36 | 0.85 ± 0.12 | 4.07 ± 0.89 | 0.733 | 0.023* |
| NGRP | nd | nd | 3.90 ± 0.00 | 3.83 ± 0.06 | nd | 0.183 | nd | nd | 6.66 ± 0.77 | 7.46 ± 0.33 | nd | 0.209 |

## Table S6. The effect of *dsbC* knockout to cell growth and protein titre (mg/L) during production of model disulphide-bond proteins in glyco-competent *E. coli* Top10F’. Statistical analysis was conducted using unpaired t-test with Welch’s correction (*P*-value < 0.05*, < 0.001***)

| **Protein models** | **Final OD_600_** | | ***P*-value** | **Protein titre (mg/L)** | | ***P*-value** |
| --- | --- | --- | --- | --- | --- | --- |
|  | ***wt*** | **Δ*dsbC*** | **wt vs Δ*dsbC*** | ***wt*** | **Δ*dsbC*** | **wt vs Δ*dsbC*** |
| RNase A | 2.69 ± 0.05 | 3.60 ± 0.00 | 8.6 x 10^-4^ *** | 2.94 ± 0.37 | 5.43 ± 0.69 | 0.011* |
| NGRP | 5.33 ± 0.09 | 4.96 ± 0.00 | 0.02* | 7.79 ± 0.42 | 7.95 ± 1.53 | 0.873 |
| svFv13R4 | 5.17 ± 0.05 | 3.15 ± 0.05 | 7.2 x 10^-7^**** | 8.68 ± 0.68 | 3.19 ± 0.43 | 7.3 x 10^-4^*** |
| scFv13R4CM | 3.84 ± 0.00 | 4.56 ± 0.08 | 0.004** | 6.52 ± 0.17 | 6.57 ± 0.69 | 0.912 |
